# Drug screening approach combines epigenetic sensitization with immunochemotherapy in cancer

**DOI:** 10.1186/s13148-019-0781-3

**Published:** 2019-12-11

**Authors:** Chiara Facciotto, Julia Casado, Laura Turunen, Suvi-Katri Leivonen, Manuela Tumiati, Ville Rantanen, Liisa Kauppi, Rainer Lehtonen, Sirpa Leppä, Krister Wennerberg, Sampsa Hautaniemi

**Affiliations:** 1 Research Program in Systems Oncology, Faculty of Medicine, University of Helsinki, PO Box 63, Helsinki, Finland; 2 Institute for Molecular Medicine Finland (FIMM), University of Helsinki, Helsinki, Finland; 3 Department of Oncology, Helsinki University Hospital Cancer Center, Helsinki, Finland; 4 Research Program in Applied Tumor Genetics, Faculty of Medicine, University of Helsinki, Helsinki, Finland

**Keywords:** Epigenetic reprogramming, High-throughput drug screening, epigenetic inhibitors, Drug resistance in cancer, DLBCL

## Abstract

**Background:**

The epigenome plays a key role in cancer heterogeneity and drug resistance. Hence, a number of epigenetic inhibitors have been developed and tested in cancers. The major focus of most studies so far has been on the cytotoxic effect of these compounds, and only few have investigated the ability to revert the resistant phenotype in cancer cells. Hence, there is a need for a systematic methodology to unravel the mechanisms behind epigenetic sensitization.

**Results:**

We have developed a high-throughput protocol to screen non-simultaneous drug combinations, and used it to investigate the reprogramming potential of epigenetic inhibitors. We demonstrated the effectiveness of our protocol by screening 60 epigenetic compounds on diffuse large B-cell lymphoma (DLBCL) cells. We identified several histone deacetylase (HDAC) and histone methyltransferase (HMT) inhibitors that acted synergistically with doxorubicin and rituximab. These two classes of epigenetic inhibitors achieved sensitization by disrupting DNA repair, cell cycle, and apoptotic signaling. The data used to perform these analyses are easily browsable through our Results Explorer. Additionally, we showed that these inhibitors achieve sensitization at lower doses than those required to induce cytotoxicity.

**Conclusions:**

Our drug screening approach provides a systematic framework to test non-simultaneous drug combinations. This methodology identified HDAC and HMT inhibitors as successful sensitizing compounds in treatment-resistant DLBCL. Further investigation into the mechanisms behind successful epigenetic sensitization highlighted DNA repair, cell cycle, and apoptosis as the most dysregulated pathways. Altogether, our method adds supporting evidence in the use of epigenetic inhibitors as sensitizing agents in clinical settings.

## Background

DNA methylation and histone modifications dynamically regulate the chromatin structure, playing an important role in defining and maintaining cells’ identity [1]. Epigenome disruption has also been linked to several diseases including cancer [2–4] and causing treatment failure [5]. Inhibitors of enzymes responsible for writing, reading, and erasing epigenetic marks can be used as cytotoxic agents on tumor cells and several of them are already in clinical trial [6]. Importantly, epigenetic compounds have recently been shown to reprogram cellular phenotypes, which enable a novel treatment approach that exploits the plastic nature of the epigenome to turn drug resistant cancer cells into sensitive ones [7, 8].

Episensitization studies so far have focused on manually testing the sensitizing potential of one or few epigenetic compounds [7, 9]. To advance the episensitization experiments to larger-scale level, we developed a novel, high-throughput drug screening protocol to enable non-simultaneous administration of multiple compounds over a period spanning several days. We also created an inhibitor collection to test all main classes of epigenetic enzymes, comprising DNA methyltransferases (DNMTs), histone methyltransferases (HMTs), histone acetyltransferases (HATs), histone demethylases (HDMs), histone deacetylases (HDACs), and bromodomains (BRDs).

We systematically assessed the ability of epigenetic inhibitors to overcome treatment resistance in diffuse large B-cell lymphoma (DLBCL). The standard of care therapy given to previously untreated DLBCL patients of all ages and subtypes is the immunochemotherapy called R-CHOP [10]. This combination consists of rituximab, cyclophosphamide, doxorubicin, vincristine, and prednisone and cures approximately 60% of the patients [10]. The key compounds in R-CHOP are the topoisomerase II inhibitor doxorubicin and the monoclonal antibody rituximab. Doxorubicin is an anthracycline that induces DNA damage, and its addition to the regimen increased the 10-year overall survival of 20% [11, 12]. Rituximab targets the B-cell surface protein CD20, and both activate immune response and induce apoptosis via p38 MAP-kinase signaling pathway [13, 14]. Addition of rituximab to CHOP increased the 5-year overall survival by 10% [15–19]. Even though several genes, such as *TP53*, *STAT3/6*, *CDKN2A*, and *EZH2*, have been suggested to confer resistance to R-CHOP [20, 21], there is no clinically effective treatment available for resistant patients. Given that DLBCL is the most common aggressive lymphoid cancer [22] and that patients who are not cured with R-CHOP have dismal prognosis, novel strategies to overcome R-CHOP resistance are urgently needed.

Our observations from screening 60 epigenetic inhibitors in four DLBCL cell lines revealed HDAC and HMT inhibitors as particularly effective in sensitizing these cell lines to doxorubicin and rituximab. Our results further show that epigenetic sensitization is achieved at lower doses than epigenetic cytotoxicity, potentially causing less severe side effects in clinical settings. Thus, the herein identified inhibitors are clinically promising candidates for combination treatment of resistant of refractory DLBCL patients.

To further describe mechanisms involved in the effectiveness of epigenetic compounds, we generated transcriptome sequencing and immunofluorescence data from DLBCL cell lines before and after treatment. Analyses of these data highlighted dysregulation of DNA repair as a key mechanism for episensitization. To facilitate exploiting our data and results, we have implemented an interactive Results Explorer tool (http://app.anduril.org/DLBCL_DSRT).

## Results

### High-throughput multi-step drug combination screening

**Fig. 1:**
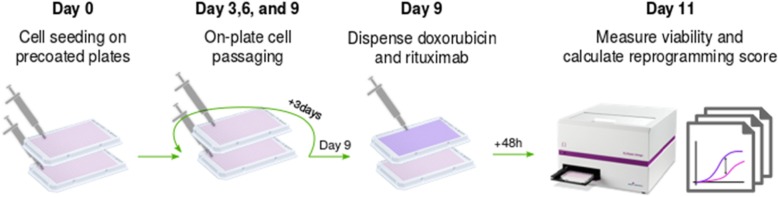
Pretreatment screening protocol to test the reprogramming activity of multiple compounds. The protocol is developed to flexibly test non-simultaneous drug combinations. In our experimental design, this included pretreatment with 60 epigenetic inhibitors, followed by treatment with rituximab and doxorubicin, the major constituents in the R-CHOP combination therapy. At day 0, cells are seeded on microplates precoated with the pretreatment compounds at five different concentrations. Cells are then passaged in the plate every third day using automated liquid handling, including the corresponding pretreatment compound in the culture media. After 9 days of pretreatment, cells are treated with a fixed concentration of doxorubicin and rituximab to compare the activity of the pretreatment alone (pink dose-response curve) vs. the activity in combination with the standard treatment (purple dose-response curve)

To systematically investigate the reprogramming ability of multiple epigenetic compounds, we have designed a high-throughput screening protocol that uses automated liquid handling to pretreat suspension cells with epigenetic inhibitors before exposing them to doxorubicin and rituximab, as representatives of standard treatment. The protocol comprises three main steps. First, cells are seeded on two replicate sets of microplates with previously administered reprogramming compounds. A 10,000-fold concentration range is used to test each epigenetic inhibitor, to determine the optimal dose inducing sensitization. Second, cells are incubated with the compounds for either 1 or 3 days (pilot experiment, Additional file 1: Figure S1), or for 9 days using on-plate passaging protocol (Fig. 1). This allows estimation of the time needed by each compound to induce cellular reprogramming.

The 9-day pretreatment is too long for cells to survive without fresh media. Thus, we developed a robotic-based protocol for on-plate cell passaging. After pretreatment, one plate set is treated with rituximab and doxorubicin while keeping another pretreated plate set as control. Third, we measured cell viability and estimated the sensitization induced by each compound using a reprogramming score, i.e., the maximum difference in cell viability between the effect of the epigenetic inhibitor alone and the effect of the epigenetic inhibitor followed by administration of rituximab and doxorubicin.

### Epigenetic inhibitors sensitize DLBCL cell lines to doxorubicin and rituximab

We applied the screening protocol to investigate whether epigenetic inhibitors are able to sensitize four DLBCL cell lines (Oci-Ly-3, Riva-I, Su-Dhl-4, and Oci-Ly-19) to doxorubicin and rituximab. We first conducted a pilot screening using short pretreatment times up to 3 days (described in the Additional file 1: Figure S3). This pilot experiment indicated that increasing the length of the pretreatment window enhances the reprogramming ability of epigenetic inhibitors. Thus, we increased the pretreatment time to 9 days in the main drug screening assay.

**Fig. 2:**
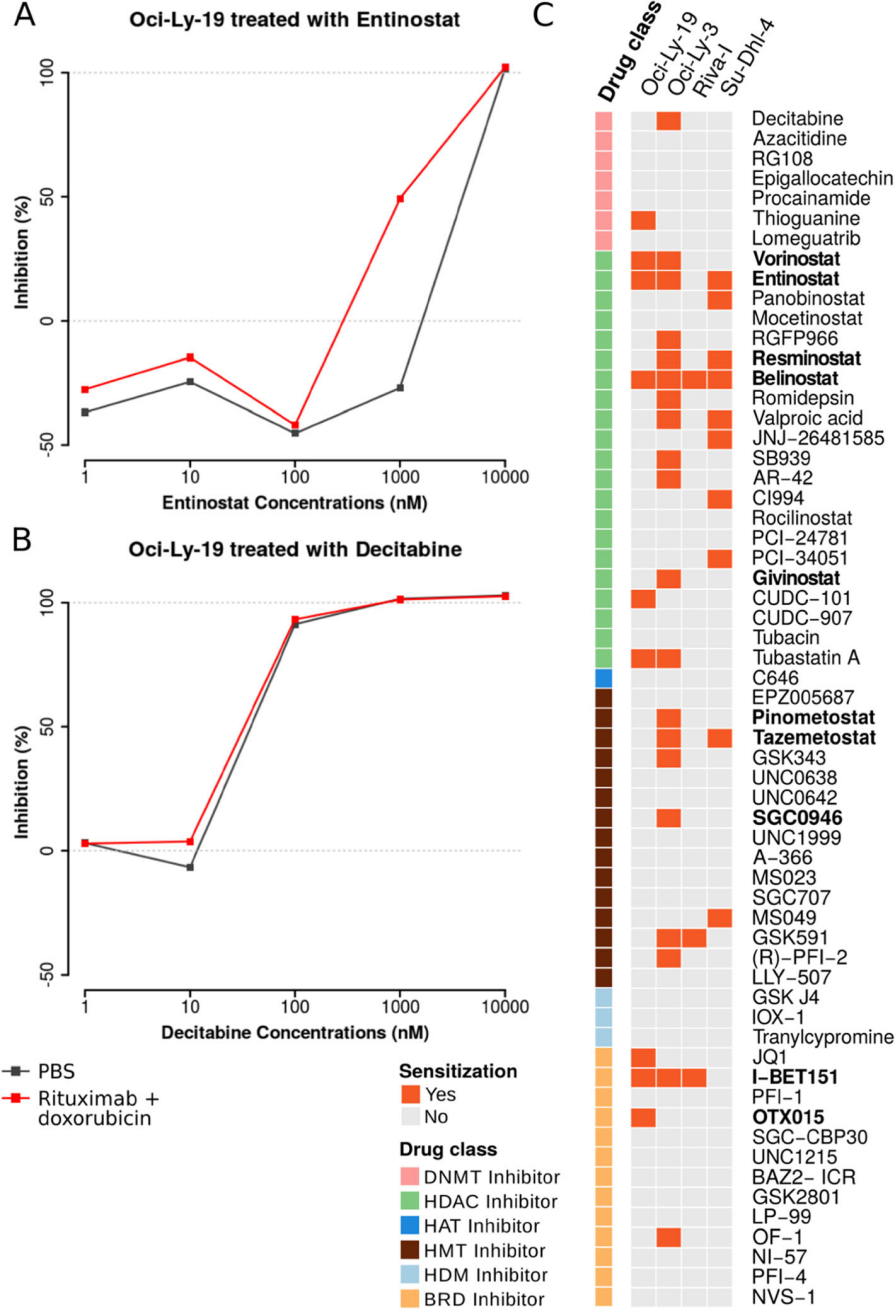
Results of the high-throughput multi-step drug combination screening with 9 days of epigenetic pretreatment. **a** An example of a compound that induced sensitization to rituximab and doxorubicin vs. phosphate-buffered saline (PBS). The combination induced 50% growth inhibition at 1000 nM concentration, which is not seen with monotreatment with entinostat. The main aim of this report is to identify such sensitizising compounds. **b** An example of a compound that does not sensitize cells to rituximab and doxorubicin, but has a cytotoxic effect. **c** Summary of the reprogramming screening hits of 60 compounds across four DLBCL cell lines. Reprogramming scores above a threshold of 30% (see Materials and Methods) and whose dose-response curve passed quality inspection are considered as hits and marked in orange. Ten compounds, marked in bold, were selected for the synergy assay based on their reprogramming potential and mechanisms of action. All dose response curves are available in Results Explorer (http://app.anduril.org/DLBCL_DSRT)

With the 9-day reprogramming, HDAC inhibitors sensitized all cell lines (Fig. 2c), while BRD and HMT inhibitors induced sensitization in three cell lines. Oci-Ly-3 cells were the most responsive, with 20 out of the 60 epigenetic inhibitors able to sensitize to doxorubicin and rituximab. Oci-Ly-19 and Su-Dhl-4 cells were sensitized by nine and 10 inhibitors, respectively. Riva-I was the most resistant cell line and was reprogrammed only by three inhibitors. Varying response to the inhibitors was expected due to heterogenous nature of DLBCL and because compounds such as HDAC inhibitors are known to have different efficacy depending on cancer type and dosage [23]. The optimal concentration at which each compound induced reprogramming was always lower than the concentration at which the same compound would induce cytotoxicity. Dose response curves are available in the Results Explorer.

### Epigenetic reprogramming acts synergistically with rituximab and doxorubicin

We conducted a drug synergy assay to validate the observed reprogramming effect of the 10 most potent inhibitors. Compounds with three or more hits, i.e., belinostat, entinostat, and I-BET151, were tested for synergy in all four cell lines, whereas the other compounds were administered only to those cell lines they reprogrammed in the screening. This validation assay followed the design shown in Fig. 1, but varying concentrations of the epigenetic inhibitors, as well as of rituximab and doxorubicin, were now used (see Additional file 1 and Additional file 2: Table S1).

**Fig. 3:**
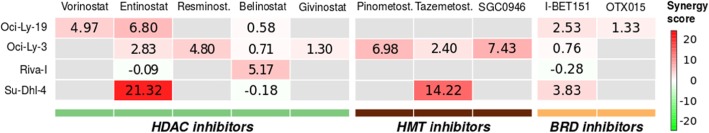
Synergy scores of the top candidate pretreatments after 9-day pretreatment. The figure shows the median scores of three replicate experiments for each measurement where higher score (red) represents synergy with doxorubicin and rituximab, and lower score (green) antagonism. For example, entinostat shows high synergy in all cell lines except Riva-I, whereas belinostat has the highest synergetic killing effect in Riva-I but less so in the other cells lines. Grey boxes represent untested combinations

The synergy plots are available through the Results Explorer, while Fig. 3 summarizes the synergy scores. A score close to zero indicates that the killing effect of an inhibitor is independent from the killing effect of doxorubicin and rituximab, whereas a high score indicates a synergistic effect [24]. None of the compounds showed high negative scores, which indicates lack of antagonistic effects. The highest synergy scores were observed in compounds targeting HDACs (vorinostat, entinostat, resminostat, belinostat) or HMTs (pinometostat, tazemetostat, SGC0946). The most potent sensitization effects were induced by the entinostat and tazemetostat. Inhibition of BRDs showed lower synergy.

This validation experiment confirmed the findings in the original screen: Oci-Ly-3 cells were the most responsive to reprogramming, and HDAC and HMT inhibitors sensitized them to rituximab and doxorubicin. Su-Dhl-4 and Oci-Ly-19 cells responded to more than one synergistic inhibitor, whereas belinostat was the only compound synergistically reprogramming Riva-I cells.

### Epigenetic sensitization to doxorubicin is achieved through reprogramming of DNA repair mechanisms

**Fig. 4:**
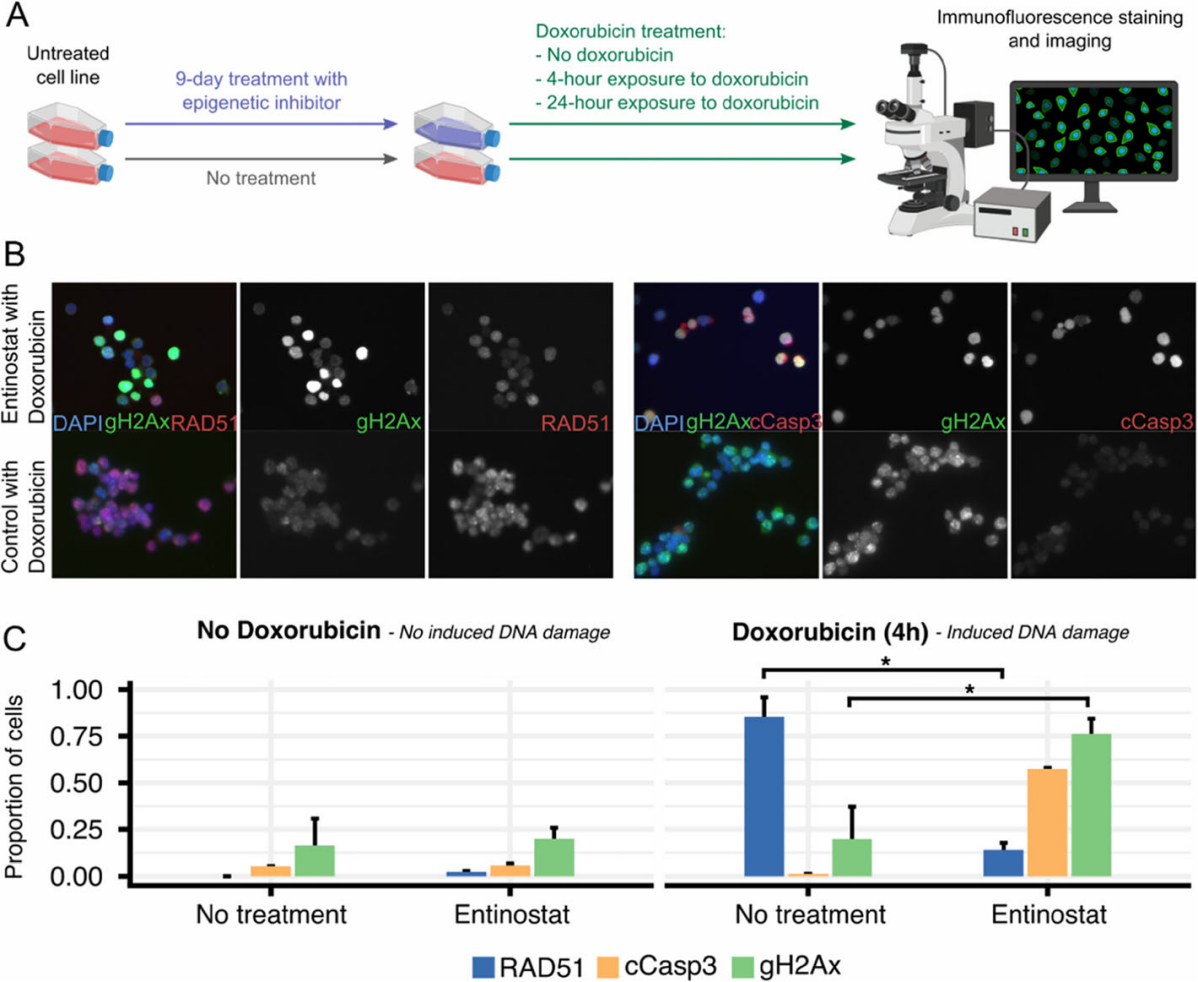
Effect of doxorubicin treatment in Oci-Ly-19 after entinostat treatment on DNA repair mechanisms quantified by immunofluorescence assay. **a** Schematic representation of the experimental procedure prior immunofluorescence staining. Cell lines were treated with epigenetic inhibitors (i.e., entinostat, belinostat, vorinostat, or tazemetostat) at days 0, 3, 6, and 9, while a copy was kept untreated as control. After 9 days, both treated and control cell lines either received no doxorubicin or were exposed to doxorubicin for 4 and 24 h. Cells were then stained using immunofluorescent antibodies and imaged. Cells not receiving any treatment served as negative control, while cells not pretreated with epigenetic inhibitors but treated with doxorubicin served as positive control. **b** Immunofluorescence images of doxorubicin treated cells after entinostat treatment (above) and untreated (below). Composite image includes also DAPI (blue). **c** Quantification of proportion of cells positive for each marker in each image. The markers shown quantify apoptosis (cCasp3), DNA damage (gH2Ax), and homologous recombination (RAD51) (see Methods). Entinostat-induced sensitization to exogenous DNA damage is detected as reduced RAD51 foci, increased cCasp3 positivity and increased gamma-H2AX positivity in doxorubicin-treated cells. Asterisks indicate significant regulation (*p* < 0.05). All measurements from the immunofluorescence assay are shown in Additional file 1: Figure S4

Enhanced DNA repair is one of the key resistance mechanisms for doxorubicin [25], so we hypothesized that the observed sensitization might be due to impaired repair. Immunofluorescence imaging of cCasp3, gH2Ax, and RAD51 is a well-established and quantitative approach for assaying DNA repair: cCasp3 is an early and specific indicator of apoptosis, gH2Ax measures the amount of DNA damage, and RAD51 indicates that homologous recombination is actively repairing DNA. Hence, we used immunofluorescence imaging of these three proteins (Fig. 4a) to investigate DNA repair pathways affected by entinostat, tazemetostat, belinostat, and vorinostat (which showed high synergy with doxorubicin and rituximab). Of note, these compounds are also clinically relevant as belinostat and vorinostat received FDA-approval for the treatment of patients with relapsed or refractory peripheral T-cell lymphoma and cutaneous T-cell lymphoma, while entinostat and tazemetostat have obtained FDA “Breakthrough” and “Fast Track” designations, respectively.

Cells treated with the HDAC inhibitors (entinostat, belinostat, vorinostat) showed reduced RAD51 foci formation (Additional file 1: Figure S4), suggesting impaired homologous recombination (HR). Non-homologous end joining (NHEJ) was upregulated in cells treated with HDAC inhibitors, which was expected as NHEJ is often seen as a compensatory effect for impaired HR. Cells treated with the HMT inhibitor tazemetostat did not show significant effect on DNA repair pathways.

These results support the hypothesis that HDAC inhibitor sensitization occurs by impairing HR repair, as shown in Fig. 4b, c. Entinostat alone does not affect the number of cells positive for double strand breaks, apoptosis, or HR, compared to the untreated control. However, the response to doxorubicin was strikingly different in cells treated with entinostat compared to untreated cells. The control cells were able to repair DNA damage due to high HR activity (Fig. 4c, blue bar) and thus avoid apoptosis.

### Transcriptomic analysis identifies disruption of DNA repair, cell cycle, and apoptosis as potential mechanisms behind epigenetic sensitization

To further characterize the molecular mechanisms affected by the observed epigenetic sensitization, we performed RNA-seq of the four cell lines before and after treating them with belinostat, entinostat, vorinostat, and tazemetostat (Additional file 1: Figure S5A). Differentially expressed genes (DEGs) between treated and untreated cells are shown in Additional file 1: Figure S5 B-E, and can be browsed in the Results Explorer. Gene expression landscape across cell lines and treatment conditions is shown in Additional file 1: Figure S6.

**Fig. 5:**
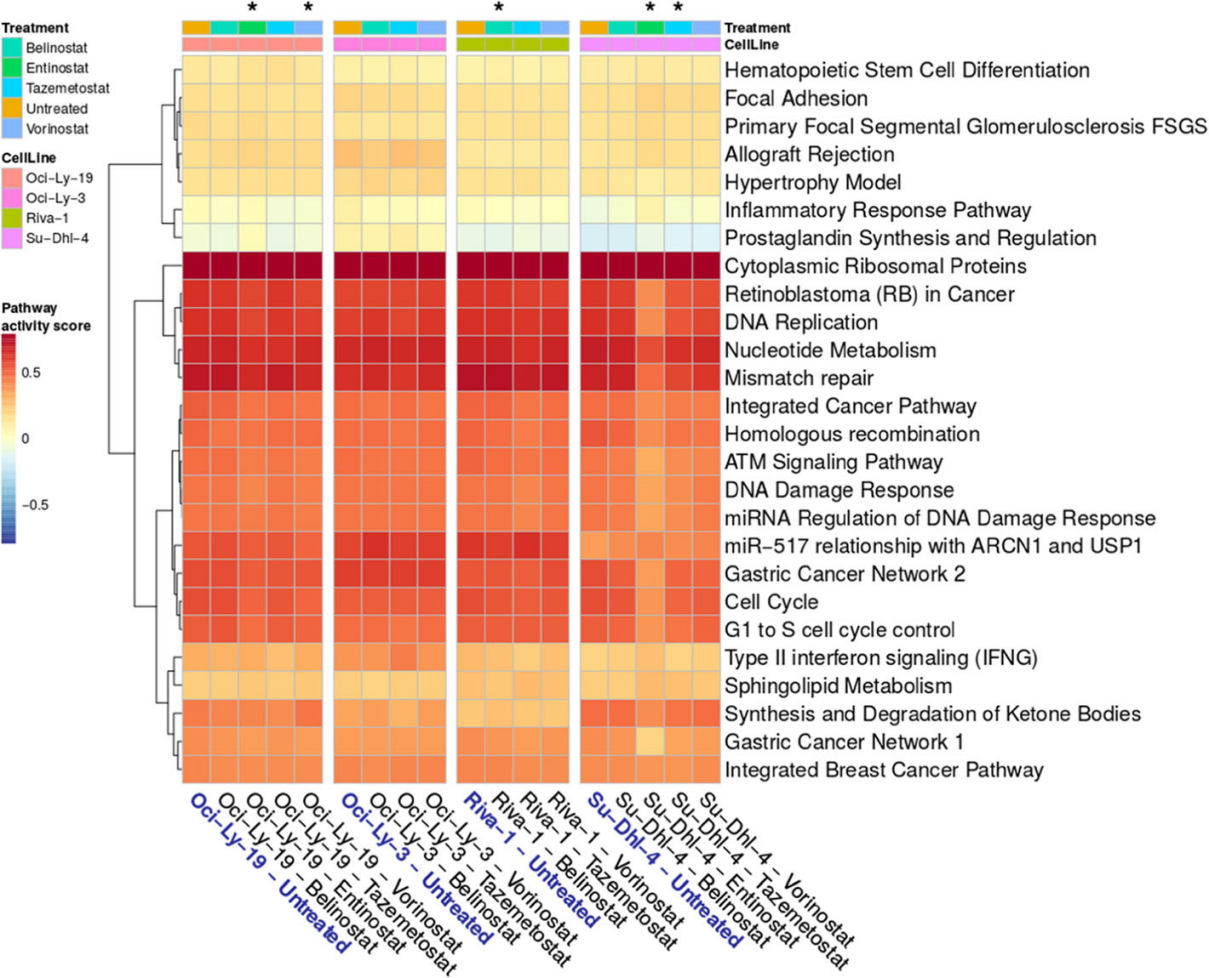
Summary of pathway analysis results for all cell lines and conditions. Columns represent the cell line–treatment combination (untreated conditions are marked in blue). Sensitizing combinations are marked by an asterisk. The rows include the top pathways identified from WikiPathways database. All pathway analysis results are available in Additional file 3: Table S2

We used DEGs from each successfully reprogrammed combination and performed pathway enrichment analysis to explore the reprogramming mechanisms. An overview of the top pathways identified using WikiPathways database is shown in Fig. 5. All pathway results including *p* values and pathway-specific DEGs for KEGG, Reactome, and WikiPathways are provided in Additional file 3: Table S2. All sensitized combinations showed changes in immune response mechanisms. This was expected since DLBCL originates from B-cells, which produce antibodies in the adaptive immune system [26]. Our analysis further revealed the major histocompatibility complex (Additional file 3: Table S2) as one of the pathways most affected by HDAC inhibitors, which is in line with a study by Eckschlager and colleagues [23].

DNA damage and repair mechanisms were dysregulated in Su-Dhl-4 and Oci-Ly-19 cells treated with entinostat, as well as in Su-Dhl-4 cells treated with tazemetostat. When comparing untreated and treated conditions for all successfully sensitized cell line and inhibitor combinations, we identified DEGs belonging to HR, NHEJ, and other DNA repair pathways (Additional file 4: Table S3). While DEGs involved in NHEJ were identified only in Su-Dhl-4 cells treated with entinostat, HR genes were differentially expressed both in Su-Dhl-4 cells treated with entinostat and tazemetostat, as well as in Oci-Ly-19 cells treated with entinostat and vorinostat. In particular, these combinations showed downregulation of *XRCC2* and *POLQ. XRCC2* is essential for the proper functioning of HR [27], and knockdown of *POLQ* in HR-deficient tumors enhances cell death [28]. Thus, decreased expression of both genes may contribute to the sensitization of doxorubicin-resistant DLBCLs. Additionally, Su-Dhl-4 cells treated with entinostat or tazemetostat also showed downregulation of *RAD51, RAD54L, BRCA2*, and three Fanconi anemia genes *(FANCA*, *FANCB*, and *FANCM). BRCA1* expression was suppressed in *Su-Dhl-4* cells in response to entinostat. In Riva-I cells, belinostat-induced differential expression did not have an impact on the expression of any HR gene, making it the only cell line reprogrammed by inhibition of HDACs and not showing transcriptional changes in DNA repair.

Disruption of cell cycle and DNA replication were also mechanisms identified in several sensitized combinations (i.e., Su-Dhl-4 and Oci-Ly-19 cells treated with entinostat, and Su-Dhl-4 cells treated with tazemetostat). In particular, treatment of Su-Dhl-4 cells with entinostat led to upregulation of *CDKN1A*, an HDAC inhibitor mechanism previously suggested to induce cell cycle arrest [23].

Other pathways identified in our analysis included cell adhesion (altered in all sensitized combinations except Riva-I cells treated with belinostat) and TGF-β signaling (disrupted in Oci-Ly-19 cells treated with either entinostat or vorinostat, and in Su-Dhl-4 cells treated with entinostat). Death receptors and ligands belonging to the TNF and TNF-receptor superfamilies were differentially expressed in all sensitized combinations. Indeed, HDAC inhibitors have been demonstrated to affect apoptosis through dysregulation of such protein families [23]. Pathway analysis also showed disruption of the apoptotic pathway in Riva-I cells treated with belinostat (Additional file 3: Table S2).

### Whole exome-sequencing suggests genomic variants relevant to drug response

To analyze the genomic variants of the cell lines, we performed whole exome sequencing. We decided to produce sequencing data directly from our cells to avoid bias due to genetic evolution of the cell lines [29]. This analysis identified somatic mutations and known germline single nucleotide polymorphisms (SNPs) that could influence drug response or the synergistic effect of epigenetic sensitizing compounds used in combination with doxorubicin and rituximab (Additional file 5: Table S4).

Among epigenetic genes, *EZH2* was found mutated (p.Y590S, missense mutation) in Su-Dhl-4, the cell line responding to the EZH2 inhibitor tazemetostat. Interestingly, this mutation has been reported to increase sensitivity to EZH2 inhibitors [30]. We also observed a truncating mutation (p.R1322X) in *CREBBP*, impairing histone acetylation, and transcriptional regulation of its targets [31] was shared in Riva-I and Oci-Ly-19 (subclonal). Truncation of *CREBBP* is acquired in relapsed acute lymphocytic leukemia [31], further supporting its important role in mediating chemotherapy resistance in lymphoid malignancies. In the same gene category, we observed a truncating mutation in the *ARID1A* gene (p.Q474X) of Riva-I cells. *ARID1A* was previously reported to encode for a critical transcription factor in the absence of HDAC6 [32]*.* It is also the most commonly mutated and functionally disrupted component of the tumor suppressor chromatin remodeling SWI/SNF complex and thereby has been reported as an important epigenetic modulator [33] and contributor to genetic and genomic instability and response to DNA damaging agents [34].

All our cell lines harbor at least one potentially functional mutation in genes encoding epigenetic enzymes targeted in this study or in genes potentially contributing to response to epigenetic inhibitory drugs reported in other studies. Notably, the majority of these genes are among the most recurrently altered drivers in DLBCL *(MYD88* in 18%, *CREBBP, ARID1A*, and *TP53* in 10%, and *EZH2* in 6% of patients) [35]. A comprehensive genomic profiling of the cell lines is reported in the Additional file 1.

### Increased CD20 expression does not sensitize all cell lines to rituximab

Next, we investigated whether rituximab sensitization in response to epigenetic modifiers resulted from increased expression of the *MS4A1* gene encoding for CD20. All four untreated cell lines express *MS4A1* (with Oci-Ly-19 showing the lowest expression), but none of the four tested epigenetic compounds was able to upregulate *MS4A1* expression. However, *CD40*, a key effector of CD20 on B cells [36], was inhibited in Oci-Ly-19 cells in response to vorinostat or tazemetostat.

We also examined if sensitization to rituximab could be due to increased expression of CD20 on the cell surface. Two compounds shown to support CD20 transport to the cell membrane were tested using our high-throughput screening (Additional file 1: Figure S7). Rifampicin, an antibiotic shown to restore efficacy of anti-CD20 antibodies [37], was successful only on Oci-Ly-19 cells. Suramin, a small molecule that inhibits CD40, sensitized the rest of the cell lines. However, since this sensitization was observed at different pretreatment times for different cell lines, we cannot conclude that upregulation of CD20 expression is required for sensitization to rituximab.

When comparing the expression of rituximab-related genes [38] across untreated cell lines, we observed that Oci-Ly-3 presented a unique transcriptomic profile. Specifically, Oci-Ly-3 was the only cell line showing overexpression of *CXCL13*, a B cell-attracting chemokine [39], and downregulation of *CD27*, a proapoptotic gene previously shown to link HDACs activation and cell cycle arrest [40].

## Discussion

Finding effective treatment options for relapsed and refractory malignancies is a major challenge in cancer therapy. Due to the plasticity of the epigenome, epigenetic inhibitors are a particularly interesting class of compounds to sensitize cancer cells to standard therapeutic options [41]. Hence, we designed a high-throughput experimental protocol that allows identifying epigenetic inhibitors able to (re)sensitize cancer cells to standard therapeutic agents. This approach is a major step towards finding clinically useful and effective drug combinations. Moreover, the customizable plate layout and the ability to vary experiment duration by on-plate cell passaging make this protocol suitable for testing multi-step delivery for other drug combinations.

As a case study, we tested our protocol on DLBCL cell lines that represent the heterogeneity of the disease. We demonstrated how this experimental approach helped to systematically evaluate the sensitization power of 60 epigenetic inhibitors. Our results indicate that most of these inhibitors require several days to effectively induce reprogramming. Only a few compounds, mainly HDAC inhibitors, were able to sensitize cell lines within 1 and 3 days of pretreatment. Increasing the pretreatment length allowed us to identify additional actionable mechanisms that would be missed with a shorter assay. For instance, DNMT inhibitors are expected to be slow acting, since passive demethylation requires several cell cycles. Pairing 9 days of pretreatment time with multiple doses of epigenetic inhibitors sensitized all cell lines to doxorubicin and rituximab, the key compounds of R-CHOP. This suggests that epigenetic reprogramming is an effective option across all molecular subtypes in DLBCL.

Importantly, this study demonstrates that epigenetic drugs induced sensitization at much lower doses than those required for cytotoxicity, suggesting that when epigenetic drugs are used to sensitize rather than kill cancer cells, they are likely to cause less severe side-effects. Based on the tested pretreatment times, we propose that epigenetic inhibitors should be administered in a reprogramming mode, i.e., several doses and days before chemotherapy, rather than simultaneously with chemotherapy.

In this study, HDAC inhibitors belinostat, entinostat, vorinostat, and resminostat, as well as HMT inhibitors tazemetostat, pinometostat, and SGC0946, were the most potent epigenetic drugs to sensitize cancer cells to doxorubicin and rituximab. Both classes of inhibitors are reported to be well-tolerated in clinical trials [23, 42, 43]. Further pre-clinical investigation of their reprogramming potential to identify the correct time and dose for epigenetic sensitization will broaden the use of these inhibitors as pretreatment options on chemo-resistant cancers.

This protocol allowed to identify the right dose and pretreatment length for multiple epigenetic inhibitors. We then used these parameters to investigate the mechanisms behind epigenetic reprogramming through transcriptome analysis. Each cell line achieved reprogramming through a different combination of altered pathways, but we identified dysregulation of DNA repair (especially HR), disruption of cell cycle, and effects on apoptosis as the main drivers of sensitization. This adds on previous observations on the mode of action of HDAC and HMT inhibitors [23, 44]. Resistance to DNA damaging agents, such as doxorubicin [45], has often been associated with upregulation of HR-mediated DNA repair. Our results show that belinostat, entinostat, vorinostat, and tazemetostat can sensitize DLBCL cells via disruption of the HR pathway. Further, sensitization by these inhibitors affected the expression of several DNA repair genes, such as *XRCC2* and *POLQ*, both downregulated in most sensitized combinations. These findings argue that epigenetic inhibitors could also revert resistance to a wider class of DNA damaging agents, such as platinum-based regimens and radiotherapy.

Belinostat, as the broadest pan-HDAC inhibitor in our collection, sensitized cells with very low evidence of HR dysregulation but strong truncation of ARID1A transcription factor, critical for the cell’s survival in the absence of HDAC6 [32]. In depth analysis of pan-HDAC effects on a different cell line cohort might highlight alternative mechanisms of action, such as disruption of apoptosis observed in belinostat-treated Riva-I.

Even though we found a clear link between epigenetic modifiers and sensitization to doxorubicin, we were unable to find the same for rituximab. The major reasons are the undefined mechanisms of action of CD20 and the absence of known regulatory pathways modulating this protein.

Through the genomic characterization of our cell lines, we were able to highlight commonly mutated genes among DLBCL patients [35] with functional and clinical relevance. This opens a line of pre-clinical biomarker investigation for future personalized sensitization therapy. The most striking mutation was identified on gene *EZH2* in the GCB cell line Su-Dhl-4, the best responder to EZH2-inhibitor tazemetostat. This EZH2 inhibitor has the highest efficacy in *EZH2*-mutated DLBCL patients belonging to the GCB subtype [46], and the mutation found in Su-Dhl-4 cells has been reported as a potential biomarker for response to EZH2 inhibitors [47]. Moreover, we observed high tazemetostat-driven dysregulation of cell cycle genes in these cells, potentially causing cell cycle arrest, a mechanism suggested by Knutson et al*.* [44]. Altogether, these results suggest that R-CHOP resistant DLBCL patients with *EZH2* mutations could benefit from tazemetostat pretreatment followed by R-CHOP re-challenge.

Our study has some limitations. First, when we created the compound library, only few HDM and HAT inhibitors were available. In our experiments, these compounds were not able to induce reprogramming, but testing a more extensive inhibitor collection might identify reprogramming mechanisms targeting HDM and HAT enzymes. Second, even though all DLBCL subtypes were represented in our study, the number of cell lines included was just enough to show the value of the proposed protocol but relatively small to propose clinical strategies or biomarkers with high confidence. Future studies can however use this protocol and increase the number of samples and drugs. Third, since our readout was cytotoxicity and not tumor immunity, the impact of tumor microenvironment could not be tested in this model; hence, the observed epigenetic reprogramming of rituximab resistance may be only partially detected. Fourth, some HDAC inhibitors have been shown to have off-target effects [48], which cannot be separated from the on-target effects in the transcriptomic analyses due to the experimental design we have employed. Such pleiotropic effects, however, do not impact our combination therapy results herein but may pose challenges for tolerability of the combination treatment and identification of specific biomarkers in future studies. Last, even though our results suggest that epigenetic inhibitors can be useful in a clinical setting, a more detailed analysis is needed to estimate optimal dose and treatment duration in vivo.

## Conclusions

Taken together, in this contribution we report a novel methodology to screen sensitization effect of non-simultaneous drug combinations. It is also among the few studies investigating the use of epigenetic drugs as sensitizing agents, instead of using them as mono- or combination therapy, and it is the first to do so in a systematic and high-throughput manner. The application of this method to overcome R-CHOP resistance further supports the use of epigenetic reprogramming in sensitizing DLBCL to standard immunochemotherapy combinations.

## Materials and methods

An overview of the main materials and methods is reported below. For a detailed description of experimental protocols and bioinformatic pipelines, see the Additional file 1.

### Compound collection

We curated the compound library by manually searching literature and providers for compounds inhibiting epigenetic enzymes. We collected both FDA-approved compounds and probes targeting DNMT (*n* = 7), HDAC (*n* = 21), HAT (*n* = 1), HMT (*n* = 15), HDM (*n* = 3), and BRD (*n* = 13). The full list of compounds used in each experiment is available in Fig. 2 and Additional file 1: Figure S3, while concentration ranges are listed in Additional file 2: Table S1.

### Cell lines

We performed our epigenetic screening on four DLBCL cell lines, representative of all DLBCL subtypes [49] and with varying response to rituximab and doxorubicin (Additional file 1: Figure S2). Su-Dhl-4 belongs to the GCB subtype [50], Oci-Ly-3 and Riva-I to the ABC subtype [50], while Oci-Ly-19 is unclassified.

### Screening procedure and parameters

Compounds were dissolved in DMSO and added to the assay plates using a Labcyte Echo 550 acoustic dispenser. The highest dose concentration was as advised by the supplier followed by four tenfold dilutions.

Plate layout design included randomized positive (benzethonium chloride, BzCl, Sigma-Aldrich) and negative (DMSO, Sigma-Aldrich) controls. Compound plates were stored under inert nitrogen gas in StoragePods (Roylan Developments) until needed. Cells were seeded using BioTek MultiFlo FX Random Access Dispenser, at 3000 cells/well (Riva-I, Su-Dhl-4, Oci-Ly-19) or 4000 cells/well (Oci-Ly-3) in 25 μL (1 and 3 days of pretreatment time) or 40 μL (9 days of pretreatment time). Cell plates were incubated in a Thermo Scientific Cytomat 10C incubator at 37°C and 5% CO_2_. Plates undergoing 9 days of pretreatment had a Labcyte microclime lid to reduce media evaporation during the incubation period. During the longest pretreatment time, cells were passaged every third day (i.e. on days 3, 6, and 9) directly in-plate with pretreatment drugs added to the new media. Since all cell lines grew in suspension, plates were spun down before passaging. In-plate passaging was then performed with a Beckman Coulter Biomek FXp pipetting device fitted with a 384 multichannel head. The BioMek FXp protocol included the following steps:

1. Aspirate 20 μL of old media from the culture plate (without touching the cells collected at the bottom of the well) and discard it.
2. Aspirate 20 μL of fresh media from the plate with drugs in media and dispense it on the cells.
3. Resuspend the cells by mixing with 20 μL volume five times.
4. Aspirate 20 μL of old media and cells from the culture plate and discard it.
5. Aspirate 20 μL of fresh media from the plate with drugs in media and dispense it on the cells.

With this procedure, roughly 3/4 of the media was exchanged while removing half of the cells from each well.

After pretreatment, half of the plates were treated with a fixed dose of rituximab (MabThera, diluted in phosphate-buffered saline (PBS). Roche) and doxorubicin (diluted in PBS. Sigma-Aldrich), while the other half received only PBS as control. The concentrations of rituximab and doxorubicin were determined through a drug combination assay and are listed in Additional file 2: Table S1. After treatment, cells were incubated for 48 h. Finally, cell viability was measured with Promega CellTiter-Glo reagent and BMG LABTECH FLUOstar Omega plate reader.

### Analysis of DNA damage, DNA repair, and apoptosis

We used an immunofluorescence assay to measure doxorubicin-induced DNA damage as double-strand breaks (detected as γH2Ax foci), efficiency of DNA repair via homologous recombination (detected as RAD51 foci) and non-homologous end joining (detected 53BP1 foci), and apoptosis (detected as cleaved-Casp3). Measurements obtained from untreated cells were compared with those from cells pretreated with belinostat, entinostat, vorinostat, or tazemetostat.

### Whole exome and RNA sequencing

All untreated cell lines were analyzed by whole exome sequencing to identify mutations linked to epigenetic sensitization. We also performed RNA sequencing for the four cell lines untreated, as well as treated with belinostat, entinostat, vorinostat, or tazemetostat, to identify transcriptomic changes induced by epigenetic reprogramming (see Additional files 1 and 6).

## Supplementary information

**Additional file 1.** Supplementary file containing additional methods and results, as well as the user guide for the Results Explorer.

**Additional file 2: Table S1.** Reprogramming scores and compound concentrations.

**Additional file 3: Table S2.** EnrichR DEGs pathway analysis results.

**Additional file 4: Table S3.** DNA repair DEGs.

**Additional file 5: Table S4.** Exome sequencing selected variants.

**Additional file 6: Table S5.** Drug targets.

**Additional file 7: Table S6.** Gene sets preloaded in the results browser.

## Data Availability

All raw fastq files from RNA-Seq and WES are available at SRA [51] (accession number PRJNA517451). All our processed results can be browsed through our Results Explorer tool (http://app.anduril.org/DLBCL_DSRT).

## Abbreviations

ABC: Activated B-cell
BRD: Bromodomain
DEG: Differentially expressed gene
DLBCL: Diffuse large B-cell lymphoma
DNMT: DNA methyltransferases
GCB: Germinal center
HAT: Histone acetyltransferase
HDAC: Histone deacetylase
HDM: Histone demethylase
HMT: Histone methyltransferase
HR: Homologous recombination
NHEJ: Non-homologous end joining
R-CHOP: Standard therapy for DLBCL including rituximab, cyclophosphamide, doxorubicin, vincristine, and prednisone
SNP: Single-nucleotide polymorphism
