# Supplementary material for: Drug screening approach combines epigenetic sensitization with immunochemotherapy in cancer
Source: Clin Epigenetics. 2019 Dec 11;11:192. doi: 10.1186/s13148-019-0781-3 (PMC6907220; doi:10.1186/s13148-019-0781-3)
Supplement: Supplementary file 1 — Additional file 1. Supplementary file containing additional methods and results, as well as the user guide for the Results Explorer. [file 13148_2019_781_MOESM1_ESM.docx]

# Supplementary File

**Drug screening approach combines epigenetic sensitization with**

**immunochemotherapy in cancer**

1. **Content of supplementary file**

Materials and Methods ………………………………………………………………… 1

1. Supplementary Figures S1 …………………………………………………………….. 6
2. Supplementary Figures S2 …………………………………………………………….. 7
3. Pilot epigenetic screening with pretreatment time up to three days …………………... 8
4. Supplementary Figures S3 …………………………………………………………….. 8
5. Supplementary Figures S4 …………………………………………………………….. 9
6. Supplementary Figures S5 …………………………………………………………….. 10
7. Supplementary Figures S6 …………………………………………………………….. 11
   - 1. Genomic profiling of DLBCL cell lines ………………………………………………. 12
8. Supplementary Table 1 …………………………………………………………….. 13
9. Supplementary Figures S7 …………………………………………………………….. 14
10. Results Explorer - User Guide ………………………………………………………… 15
    - 1. References …………………………………………………………………………….. 18
      2. **Additional supplementary tables as external files**
      3. - Table S1. Reprogramming scores, synergy scores and compound concentrations. (Excel)
11. - Table S2. Pathway enrichment results using EnrichR. (Excel)
12. - Table S3. DEGs belonging to DNA repair pathways. (Excel)
13. - Table S4. DLBCL cell line exome sequencing key somatic mutations and germline polymorphisms with annotations. (Excel)
14. - Table S5. Target genes of drugs used in the synergy experiment. (Excel)
15. - Table S6. Gene sets preloaded in the result explorer. (Excel)

**Materials and Methods**

- - 1. *Cell culture conditions*

Riva-I, Su-Dhl-4 and Oci-Ly-19 were cultured in RPMI medium with 10% FBS, and Oci-Ly-3 was cultured in IMDM medium with 20% FBS. Cells were passaged every second day with a ratio of 1:2 (Su-Dhl-4, Oci-Ly-3) or 1:3 (Riva-I, Oci-Ly-19). Cells were kept in an incubator at a constant 37°C and 5% CO_2_. All cell lines were authenticated by STR analyses and tested negative for mycoplasma contamination.

*Screening procedure and parameters*

Compounds were dissolved in DMSO and added to the assay plates using a Labcyte Echo 550 acoustic dispenser. The highest dose concentration was as advised by the supplier followed by four 10-fold dilutions. Details on each compound’s individual dose can be found in Table S1. Plate layout design included randomized positive (benzethonium chloride, BzCl, Sigma-Aldrich) and negative (DMSO, Sigma-Aldrich) controls. Compound plates were stored under inert nitrogen gas in StoragePods (Roylan Developments) until needed. Cells were seeded using BioTek MultiFlo FX Random Access Dispenser, at 3000 cells/well (Riva-I, Su-Dhl-4, Oci-Ly-19) or 4000 cells/well (Oci-Ly-3) in 25 $\mu$L (1 and 3 days of pretreatment time) or 40 $\mu$L (9 days pretreatment time). Cell plates were incubated in a Thermo Scientific Cytomat 10C incubator at 37$^{\circ}$C and 5% CO_2_. Plates undergoing 9 days of pretreatment had a Labcyte microclime lid to reduce media evaporation during the incubation period. During the longest pretreatment time, cells were passaged every third day (*i.e*. on day 3, 6 and 9) directly in-plate with pretreatment drugs added to the new media. Since all cell lines grew in suspension, plates were spun down before passaging. In-plate passaging was then performed with a Beckman Coulter Biomek FXp pipetting device fitted with a 384 multichannel head. The BioMek FXp protocol included the following steps:

1. Aspirate 20 $\mu$L of old media from the culture plate (without touching the cells collected at the bottom of the well) and discard it.
2. Aspirate 20 $\mu$L of fresh media from the plate with drugs in media and dispense it on the cells.
3. Resuspend the cells by mixing with 20 $\mu$L volume five times.
4. Aspirate 20 $\mu$L of old media and cells from the culture plate and discard it.
5. Aspirate 20 $\mu$L of fresh media from the plate with drugs in media and dispense it on the cells.

With this procedure roughly $3/4$ of the media was exchanged while removing half of the cells from each well.

After pretreatment, half of the plates were treated with a fixed dose of rituximab (MabThera, diluted in PBS. Roche) and doxorubicin (diluted in PBS. Sigma-Aldrich), while the other half received only PBS as control. The concentrations of rituximab and doxorubicin were determined through drug a combination assay and are listed in Table S1. After treatment, cells were incubated for 48 h. Finally, cell viability was measured with Promega CellTiter-Glo reagent and BMG LABTECH FLUOstar Omega plate reader.

*Cell lines response to rituximab and doxorubicin*

Rituximab and doxorubicin were selected because they are the main contributors of R-CHOP. The static concentration of the combination of doxorubicin and rituximab was selected by performing a drug combination assay. To emulate the conditions of the cell culture at the moment of treatment in the screening procedure, this assay followed the same protocol without pretreating with epigenetic inhibitors. On treatment day the cells are treated with combinations of seven 10-fold dilutions of doxorubicin and rituximab starting at 1000mM and 10mg/mL respectively. The plate layout included triplicates for each combination dose.

- - 1. *Microplate reader data processing*

All dose response analyses were implemented within the Anduril framework [1]. Quality control and filtering was done using positive (BzCl, cell killing) and negative (DMSO) controls. This step comprises analyzing mean, standard deviation, coefficient of variation (CV), signal to background ratio, Z’-factor, and strictly standardized mean difference (SSMD) [2]. Plates with a CV higher than 20% were discarded. Each plate was normalized using the mean of negative controls as 0% inhibition and the mean of positive controls 100%.

- - 1. *Reprogramming scores*

The reprogramming score for a pair, pretreatment and cell line, is a score between 0 and 100 defined as follows:


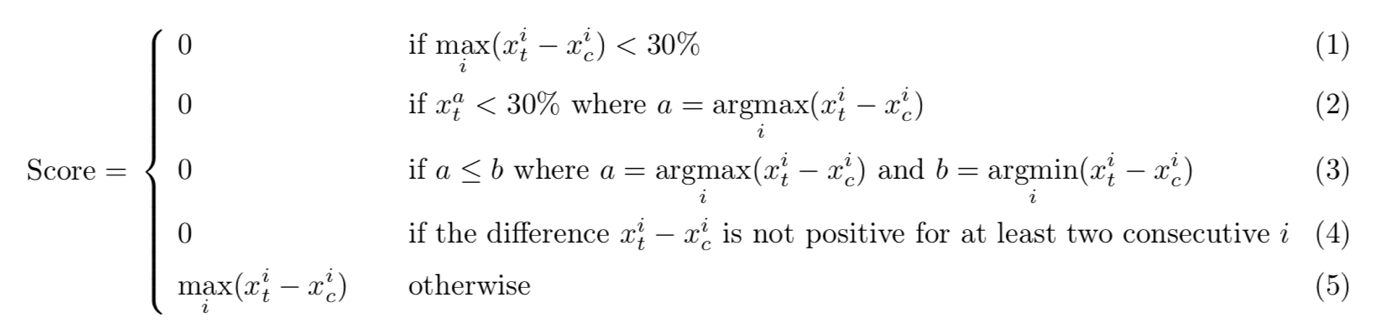


where $i$ can be any of the five doses of pretreatment compound tested, and $x_{t}$ and $x_{c}$ are the values of normalized inhibition observed in the treatment plate (the plate that was additionally treated with rituximab and doxorubicin after pretreatment time) and the control plate (the plate that only received the pretreatment) respectively. In brief, the score is defined as the highest difference between treatment and control dose-response curves, $\max_{i}(x_{t}^{i}-x_{c}^{i})$, unless it is truncated to zero. This truncation was added to ensure that the scores represent actual reprogramming events and not the effect of outliers. The algorithm returns 0 if (1) the highest difference is not large enough to consider a relevant increase on cytotoxicity, (2) despite showing reprogramming effect the inhibition achieved is low, (3) the dose that achieves the highest difference is not larger than the dose that achieves the minimum difference, (4) the reprogramming effect is not consistent throughout the dose-response curve, e.g. due to outliers. Additionally, all dose-response curves were inspected manually to confirm the quality of the data. The standard method of transforming dose-response curves with a sigmoidal function does not apply to these data due to effects caused by epigenetic reprogramming, such as enhanced cell growth, therefore a partial function was needed to determine the compounds that effectively induce epigenetic sensitization.

- - 1. *Synergy assay between epigenetic pretreatment and doxorubicin-rituximab combination therapy*

The pairs, epigenetic compound and cell line, to be validated in this assay were selected from the results shown in Figure 2 based on reprogramming scores combined with manual inspection of dose-response curves. The plate design included all combinations of epigenetic pretreatment with rituximab and doxorubicin using five concentrations for each compound and five concentrations for rituximab and doxorubicin as a combination matrix layout. Three replicates of each matrix were included in different locations in the plate. The dose ranges are detailed in Supplementary Table S1.

Synergy scores were calculated with the synergyfinder tool [3] applying the zero interaction potency model (ZIP), which compares the change in inhibition of the dose-response curves between individual drugs and their combinations, the final score quantifies the deviation from the expected inhibition in the case of zero interaction [4]. The scores are available in our Results Explorer (see Data and material availability) under “Synergy Investigation”.

- - 1. *DNA and RNA extraction and sequencing*

DNA and RNA extraction were performed using NucleoSpin Tissue (Macherey-Nagel) and NucleoSpin RNA Plus (Macherey-Nagel) respectively.

WES target enrichment was done with SureSelect Human Exome V5 baits (targeting 50 Mb of exonic regions in 31,522 genes) and 350 bp inset size libraries were constructed with SureSelect XT library kit (Agilent Corporation, CA, USA) according to manufacturer’s protocols.

The amount, concentration and integrity of the RNA samples were estimated with Bioanalyzer 2100 (Agilent) at the Biomedicum Functional Genomics Unit (Helsinki, Finland). Total RNA fragments (140-160 bp) including coding and long non-coding RNAs were sequenced using a strand specific protocol similar to TruSeq Stranded Total RNA (Illumina) including removal of ribosomal RNA with Ribo-Zero™ Magnetic Kit and RNaseH (Illumina) and random hexamer primers.

DNA and RNA samples were sequenced by BGI Genomics Co., Ltd. (Hong Kong) with Illumina HiSeq4000 sequencer and chemistry (Illumina Inc. CA, USA) using a standard paired-end protocol with 100 bp read length. In total, 150 Gb of clean data to reach 50x target coverage in WES and 60 million 100 bp reads for whole transcriptome analyses in RNA-Seq were produced from each sample.

- - 1. *Whole Exome Sequence analysis*

All analyses were performed by custom bioinformatics pipelines within the Anduril framework[1] to automate all steps. Quality control was done with FastQC [5] and read ends trimmed by trimmomatic [6]. After discarding low quality reads, and single strand reads, 75% of the reads from each sample were kept. Sequence alignment to reference genome hg19 was done with Burrows-Wheeler Aligner [7] and sorted with Picard tools (Broad Institute), 90% of the targets had coverage >10x for all the cell lines (average 33-34x). Variant calling and variant filtering were performed following GATK recommended practices [8] and Annovar [9]. A preliminary filter kept only splicing and exonic variants with VAF higher than 20% in at least one cell line, CADD score higher than 10 and COSMIC [10] annotation except in cases where no SNPdb annotation was available. Then all variants within genes with FPKM less than 1 in our RNA-seq data were discarded. The following additional filtering steps were performed to classify relevant variants as the cell lines do not have matched normal control. We defined a variant as somatic if it was present in two or less cell lines and had Minor Allele Frequency (MAF) less than 1% given by Annovar MaxPopFreq to minimize the chance of misclassification. Variants with MAF up to 5% were also considered somatic in entries with over six occurrences reported as somatic in COSMIC. The rest of the variants were assessed as germline. We filtered germline variants based on potential association to drugs tested for synergy with epigenetic inhibitors and if they were supported by public databases or literature. Moreover, variants annotated in ClinVar [11] were retained for further evaluation. In general, clinical relevance and drug-gene/drug-variant interactions were annotated using the following data sources: CIVIC [12], DGIdb [13], DrugBank [14], and PharmGKB [15]. Cancer driver status and drug response association of all protein alterations were predicted using Cancer Genome Interpreter [16]. Variants of selected genes were further annotated using gene-specific data bases, LOVD3 [17] for *BRCA1* and *RAD51*, and IARC for *TP53* [18].

- - 1. *RNA-seq analysis*
       - 1. Paired-ended fastq reads were preprocessed using an Anduril pipeline that combines state-of-the-art tools. Read quality before and after trimming was accessed using FastQC [19]. Adaptor removal and trimming were carried out with trimmomatic [6] using the following parameters: headcrop = 10, slidingWindow = 5:20, minQuality = 30, trailing = 30. Since for each sample more than 70% of all reads had quality above 30 and were more than 20 base pairs long after the trimming step, no dataset was discarded. Alignment was performed using two-pass STAR [20]. Reads were aligned to reference genome hg19 and the software was run using the default STAR parameters. Gene expression was then quantified using eXpress [21]. Genes not expressed in both the treated or untreated condition (*i.e.,* log2 FPKM < 1) were excluded from the analysis. Normalization and rlog transformation of gene count data was computed with the R package DESeq2 [22], and normalized data were used to compute differential expression. Log2 fold change and absolute difference where computed between the untreated and treated pairs for a total of 14 comparisons. Genes with (*i*) absolute log2 fold change > 1 and (*ii*) absolute difference > 1 were classified as differentially expressed genes (DEGs). These threshold were selected to ensure that differentially expressed genes would at least double/halve their expression when treated with epigenetic inhibitors (condition *i*), and that the gene would reach a baseline expression in at least the treated or untreated sample (condition *ii*).
         2. Due to the experimental design of this study and the absence of replicates, no p-value could be computed. We checked if any of the DEGs in such sets included any DSBs DNA repair genes. We also performed pathway enrichment for the DEGs belonging to one of the five sensitized combinations (Oci-Ly-19 entinostat, Oci-Ly-19 vorinostat, Riva-I belinostat, Su-Dhl-4 entinostat, and Su-Dhl-4 tazemetostat). We repeated this analysis using both all DEGs identified in each combination as well as those subsets of DEGs shared by all cell lines sensitized by each inhibitor but absent in those not sensitized (see sets highlighted in yellow in Figures S5B-E). The R package EnrichR [23] was used to perform pathway enrichment using the 2016 version of KEGG [24], WikiPathways [25], and Reactome [26] as reference databases. Pathways with at least three genes differentially expressed and with p-value less than 0.05 are listed in Table S2. To provide an overview of the top pathways identified in this analysis, the R package GSVA [27] was used to estimate the activity score of the pathways from WikiPathways with adjusted p-value less than 0.5, plotted in Figure 5 using the R package pheatmap.
    2. *Analysis of* *DNA damage, DNA repair and apoptosis*

Cells were cultured in T-25 flasks and treated with the epigenetic compounds for nine days. Untreated cells were used to assess endogenous levels of DNA damage and apoptosis. To induce DNA damage, cells were then exposed to 1$\mu$M (Su-Dhl-4, Oci-Ly-19, Oci-Ly-3) or 100 $\mu$M (Riva-I) of doxorubicin. Cells were collected after 4 and 24 h, centrifuged, washed with PBS, fixed with 2% buffered paraformaldehyde and spread on coated microscope slides for immunostaining. Primary antibodies against γH2Ax (ab22551, Abcam), RAD51 (sc-8349, Santa Cruz Biotechnology) and cleaved caspase 3 (9664, Cell Signaling Technology) were used to detect DNA damage, homologous recombination-mediated DNA repair and apoptosis, respectively, as previously described [28]. Images were acquired on a Nikon Eclipse-90i epifluorescence microscope. Image segmentation and quantification were performed using a custom script in the Anima framework [29], followed by statistical summary in R; mean, standard deviation, standard error, and confidence intervals (Figure S4).

- - 1. *Results Explorer*

The results explorer was created using the R package Shiny [30]. Plotly [31] was used to produce the interactive plots in the “Reprogramming Screening” and “Synergy Investigation” tabs, while the heatmaps in the “Gene Expression” and “Mutations” tabs were created using heatmaply [32]. The full list of genes included in the preloaded sets are available in Table S6.


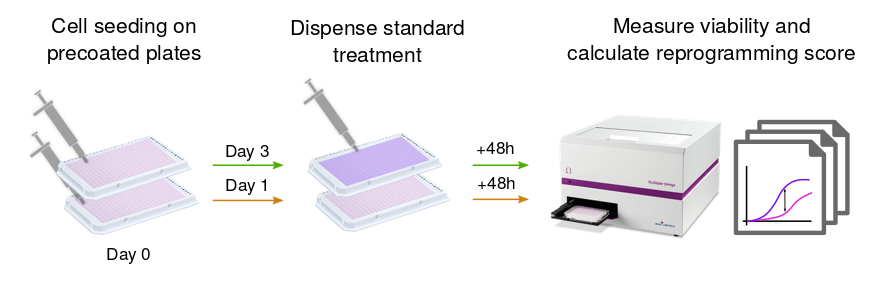


**Supplementary Figure S1:** Pretreatment screening protocol designed to simultaneously test the reprogramming activity of 44 epigenetic inhibitors. Lymphoma cells are seeded on microplates precoated with the pretreatment compounds at five different concentrations. After the pretreatment time (1 or 3 days), cells are treated with a fixed concentration of doxorubicin and rituximab in order to compare the activity of the pretreatment alone (pink dose-response curve) vs. the activity in combination with the standard treatment (purple dose-response curve).


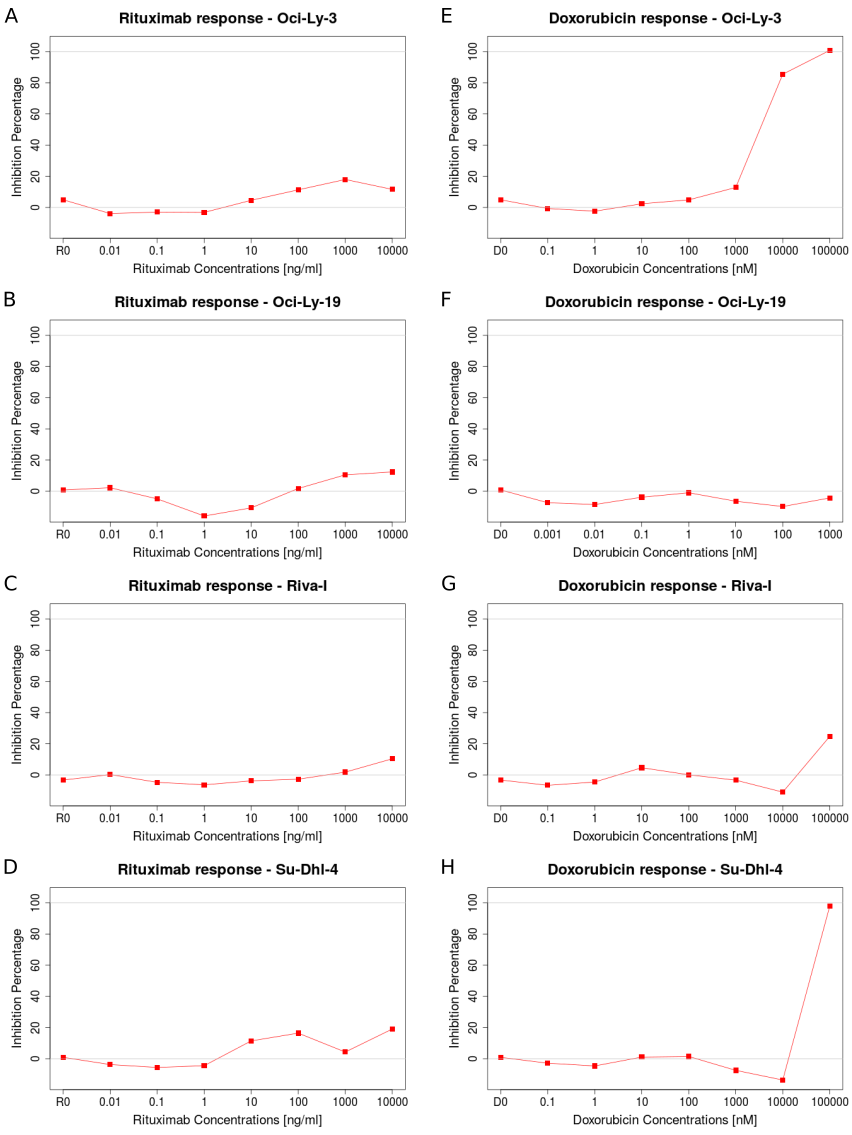


**Supplementary Figure S2:** Dose responses showing the level of rituximab (A-D) and doxorubicin (E-H) resistance of each cell line. R0 and D0 represent measurements of cell viability in the absence of rituximab and doxorubicin respectively.

- - 1. *Pilot epigenetic screening with pretreatment time up to three days*

When developing our screening protocol, we first conducted a pilot screening (Figure S1) with short pretreatment times (one and three days) to test 44 inhibitors targeting DNMT (*n* = 7), HDAC (*n* = 21), HAT (*n* = 1), HMT (*n* = 6), HDM (*n* = 3), and BRD (*n* = 6) (Table S1). Sensitization was estimated by computing a reprogramming score (Table S1) for each combination of compound and cell line (see Material and Methods). Dose response curves for this pilot experiment can be found in the result explorer, while Figure S3 summarizes which compounds successfully induced sensitization in our cell lines.

Pretreating the cells for one day induced sensitization mainly in Su-Dhl-4 cells. However, most of the potential hits occurred at low doses. Since such effect was lost when increasing the pretreatment time, we decided to further investigate it with a second screening including compounds AR-42, belinostat, CUDC-101, panobinostat, resminostat, RGFP966, rocilinostat, SB939, and tubacin. We also added mocetinostat and GSK J4 to this validation, since they were the two compounds showing reprogramming effect. Each inhibitor was screened at nine concentrations and four replicates for each dose. Only AR-42, mocetinostat, and GSK-J4 induced reprogramming in this second screening. We did not observe any sensitization for the other compounds and had to consider the low dose reprogramming as a measurement artifact, probably due to plate effect.

Three-day pretreatment resulted in the successful reprogramming of Oci-Ly-19, Riva-I, and Su-Dhl-4 cells, mainly by HDAC inhibitors. As most of the inhibitors were more effective after a 3-day pretreatment, we hypothesized that an even longer pretreatment time might increase the reprogramming effect. Hence, we decided to design our main screening assay with a 9-day pretreatment period, and we extended our drug collection to 60 inhibitors.


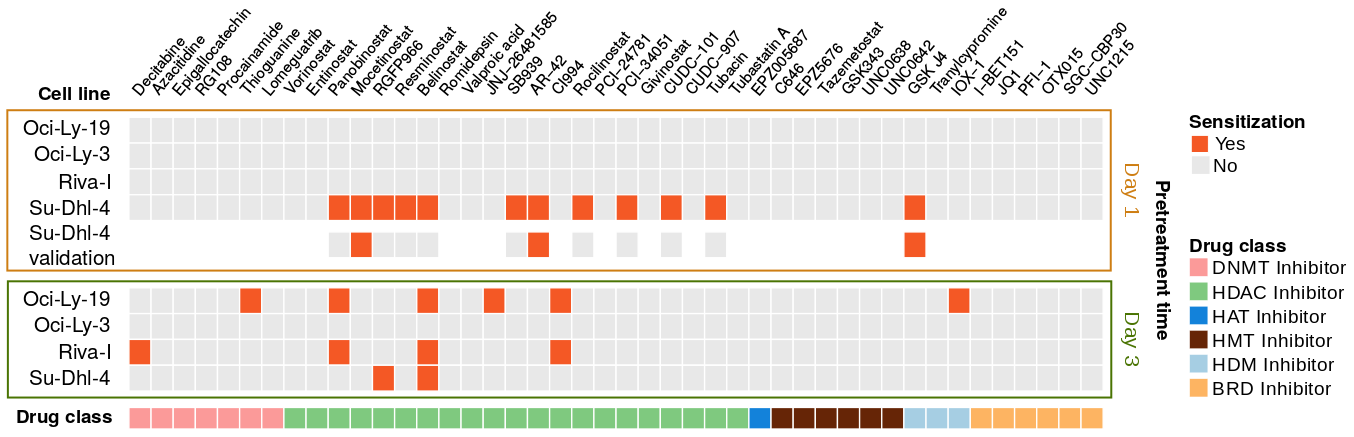


**Supplementary Figure S3:** Reprogramming screening hits. Reprogramming scores above a threshold of 30% (see Material and Methods) and whose dose-response curve passed visual inspection are considered as hits and marked in orange. Su-Dhl-4 validation row shows the results of the low dose effect assay.


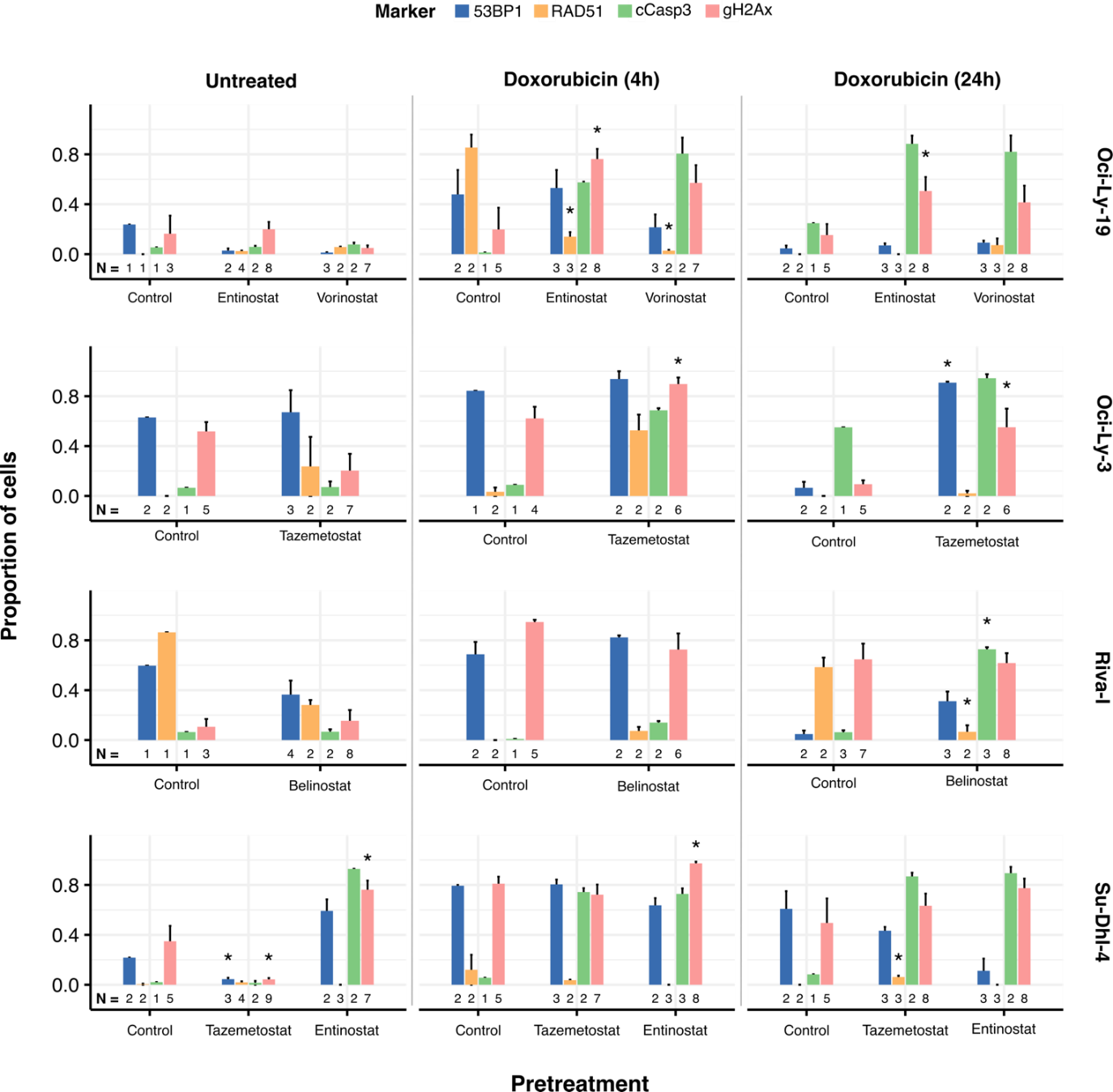


**Supplementary Figure S4:** Effect of doxorubicin treatment after epigenetic pretreatment on homologous recombination and DNA damage quantified by immunofluorescence assay. The markers 53BP1, RAD51, cCasp3, and gH2Ax represent non-homologous recombination DNA repair activation, homologous recombination DNA repair, apoptosis, and double strand DNA breaks respectively. The bar plots show doxorubicin effects on the protein expression on treated (marked with the corresponding inhibitor) and treatment-naive cells (Control), as well as epigenetic inhibitor effects in the absence of doxorubicin (left column marked as Untreated). Asterisks represent measurements significantly different from their respective Control in the cases where the number of images available (N) was sufficient for statistical test.

1.
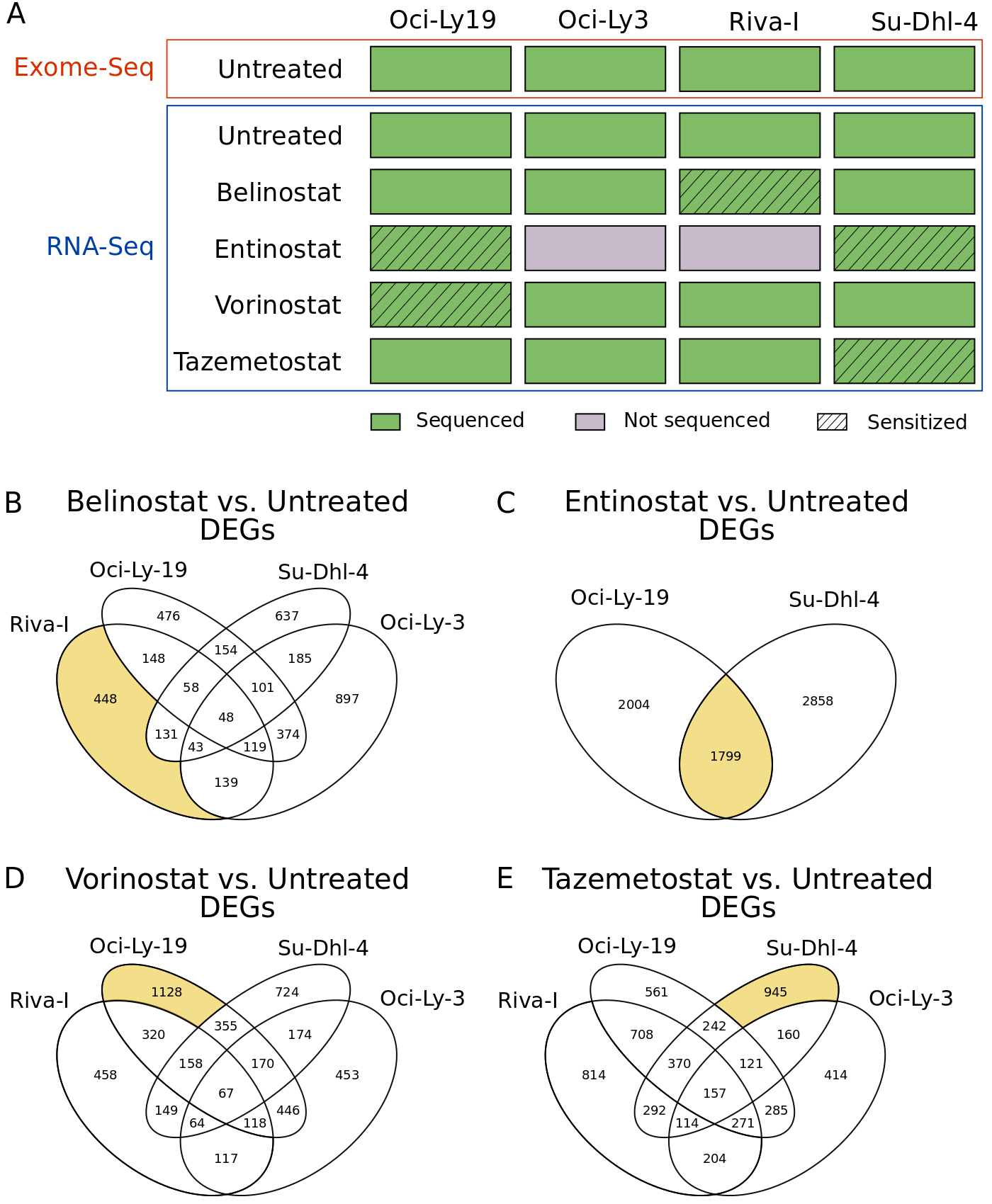

2. **Supplementary Figure S5:** Overview of sequencing data and selection of DEGs. (A) Summary of the sequencing experiments carried out in the study. Oci-Ly-3 and Riva-I cells treated with entinostat where not sequenced because the selected dose of entinostat was too toxic for these cell lines. (B-E) Venn diagrams showing the amount of DEGs induced by each epigenetic inhibitor. The portions of the diagram in yellow represent the DEGs of interested because they are found only in the sensitized combinations (B, D, and E) or are shared among the sensitized combinations (C).
3.
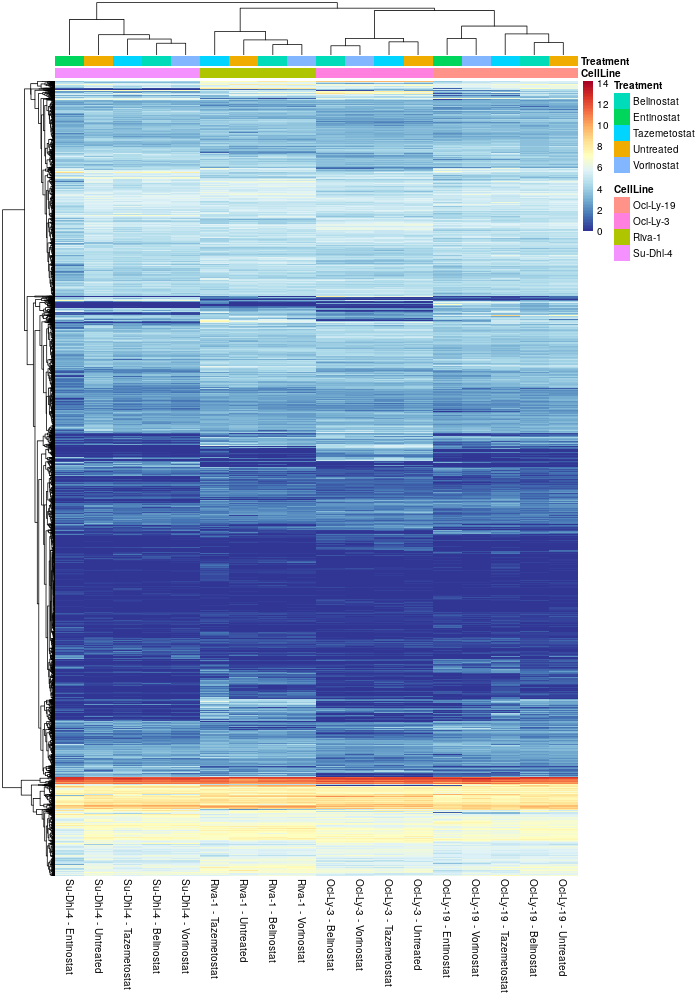

   - 1. ***Supplementary Figure S6:*** *Overview of gene expression across all cell lines. The heatmap shows the log2 fpkm values of 13,855 protein coding genes having a standard deviation (computed across all conditions) greater than 0.1.*
     2. *Genomic profiling of DLBCL cell lines*

To characterize the relevance of the identified variants, we used functional prediction and database annotations to identify genes targeted by the drugs included in our analysis (listed in Table S5). Table S4 sheet “Annotated subset” contains detailed information about manually curated and annotated somatic point mutations (*n* = 14) and germline polymorphisms (*n* = 7) with supporting functional or clinical relevance, while the “Filtered variants” sheet lists all 288 variants passing our filtering criteria, of which 268 were classified as somatic and 20 as germline. Here we discuss genes potentially involved in the observed response to epigenetic inhibitors by exploring first somatic mutations on epigenetic genes and reported drug targets, followed by preexisting germline variants that may predispose the cell lines to respond differently to compounds in our collection. Genes somatically mutated or carrying functionally relevant germline polymorphisms in pathways relevant for drug response in our study are summarized in Table 1.

Among genes targeted by the epigenetic inhibitors used in this study, we found *BCL6* (epigenetic regulation) harboring a somatic missense mutation in Oci-Ly-3, and *STAG2* which contains a truncating substitution, resulting in consistently reduced expression, in Riva-I. STAG2 is a central member of the Cohesin complex and if inactivated by such mutation, it causes genome instability and aneuploidy [33]*.* Knock-down of STAG2 is suggested to sensitize pancreatic ductal carcinoma to chemotherapy [34]*.* We also detected likely pathogenic mutations in doxorubicin related genes [15] including *TP53*, *AKT1*, and *EZH2*, and targets of HDAC inhibitors: *TP53*, *EZH2* and *MYD88*. Other genes with known germline polymorphisms associated with response to DNA damaging agents commonly used or clinically tested in DLBCL include *NQO1* (rs1800566, linked to response to alkylating agents such as cyclophosphamide, ifosfamide, and platinum compounds), *FGFR4* (rs351855, related to doxorubicin, cyclophosphamide, and fluorouracil response), and *DPYD* (rs1801160, associated to response and rs2297595 to toxicity in 5-fluorouracil treated patients). Notably, *DPYD* is directly regulated by *EZH2* and repression of DPYD through this mechanism promotes resistance and predicts poor survival in 5-fluorouracil treated patients [35].

Among the genes involved in DNA damage repair, *TP53* harbored mutations (p.E162X in Riva-I cells, and p.R141C in Su-Dhl-4 cells with VAF 1, indicating loss of heterozygosity) and a functionally unconfirmed subclonal *TP53* mutation p.K93R/p.K132R was detected in Riva-I and Oci-Ly-19 (subclonal) cells, while no *TP53* mutations were found in Oci-Ly-3 cells. *CIC*, a novel transcriptional target of mutant *TP53* and a negative tumor prognostic marker [36], was also mutated in Riva-I and Oci-Ly-19 cells. *XRCC3* [37] and *ERCC4* [38] are both involved in HR repair and the latter gene also in base excision repair, while *BCL6* is a target of AICDA driven hypermutability process [39]. All cell lines except Riva-I displayed a germline risk allele (rs861539) located in the conserved RAD51 domain of *XRCC3*, which has been previously associated to decreased DNA repair capacity in combination with variants in other HR genes [37]. The G-allele at rs1800124 in *ERCC4* detected in Riva-I has been linked to weaker DNA-protein binding resulting in decreased DNA repair [40].

| Gene Classification | | Oci-Ly-19 | Oci-Ly-3 | Riva-I | Su-Dhl-4 |
| --- | --- | --- | --- | --- | --- |
| Drug targets or reported association | **Doxorubicin** | *DPYD*********, NQO1******** | *-* | *AKT1, TP53* | *DPYD*********, FGFR4*********, NQO1*, TP53* |
|  | **Rituximab** | *DPYD** | *-* | *CREBBP* | *DPYD******** |
|  | **Tazemetostat** | *-* | *-* | *-* | *EZH2* |
|  | **Histone deacetylase inhibitors (HDACi)** | *-* | *MYD88* | *TP53* | *EZH2, TP53* |
| Epigenetic | **Histone acetyltransferases (HATs)** | *-* | *-* | *CREBBP* | *-* |
|  | **Histone methyltransferases (HMTs)** | *-* | *-* | *-* | *EZH2* |
|  | **Epigenetic regulation** | *DPYD******** | *-* | *-* | *DPYD******** |
| Chemoresponse associated cell mechanisms | **Cell cycle** | *-* | *-* | *CREBBP, STAG2, TP53* | *TP53* |
|  | **DNA repair** | *XRCC3******** | *XRCC3******** | *ERCC4*********, TP53* | *TP53, XRCC3******** |
|  | **Transcription factors** | *CIC* | *BCL6* | *ARID1A, CIC* | *RCOR1* |

***Supplementary Table 1. Observed genomic variations of interest in genes potentially contributing to response to epigenetic inhibitory drugs****. Summary of genes carrying functionally annotated somatic mutations and germline polymorphisms* shown in Table S4 sheet “Annotated subset”. These genes have been previously reported as relevant drug targets, epigenetic enzymes, or belong to pathways identified in our analysis.*


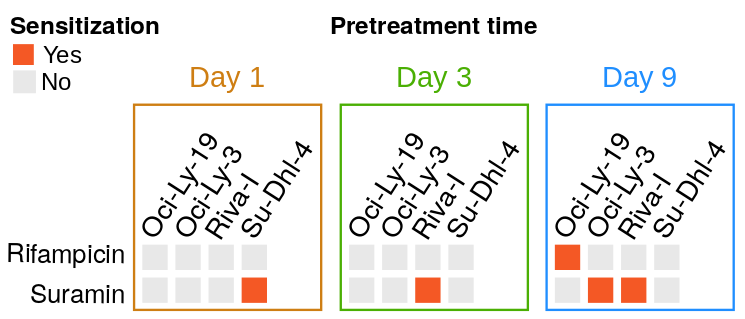


**Supplementary Figure S7:** Sensitizing effect of CD20-transport associated compounds.

**Results Explorer - User Guide**

- - - - 1. **Reprogramming screening**


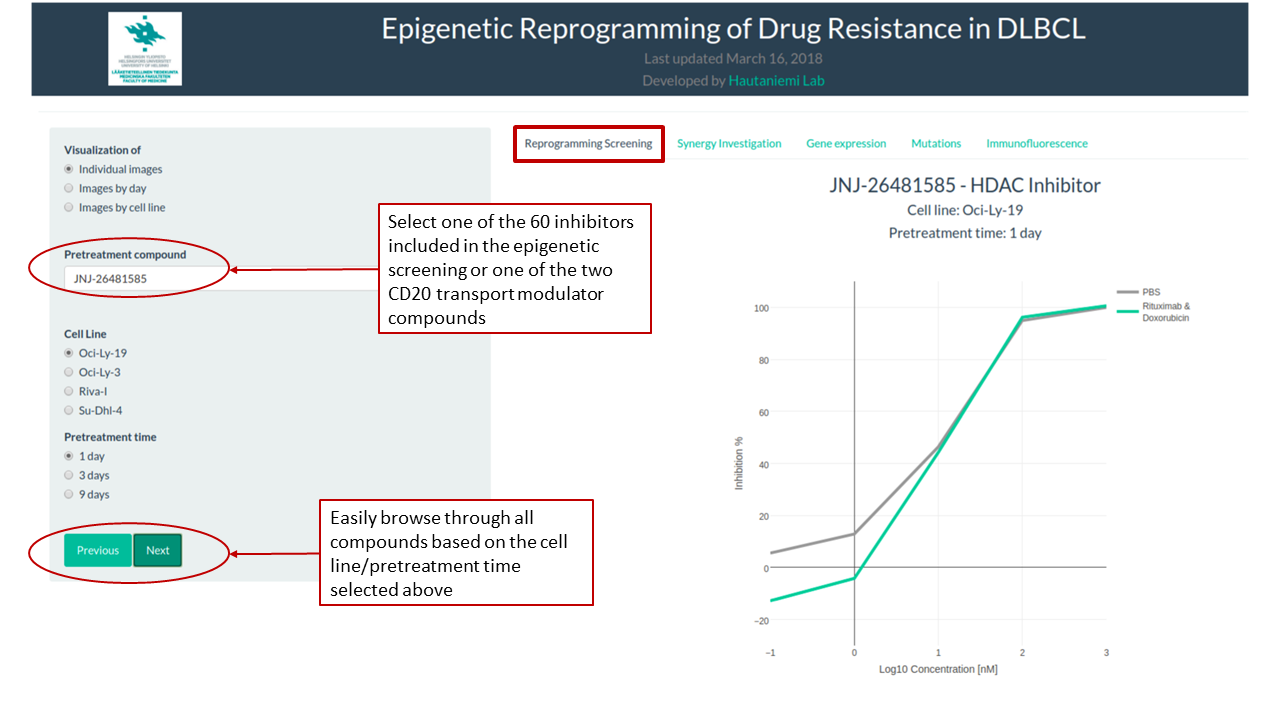
The "Reprogramming Screening" tab shows the results of the assay with five concentrations of the epigenetic inhibitors and one fixed concentration of rituximab and doxorubicin. Results for each epigenetic compound can be grouped by length of pretreatment or by cell lines for comparisons.

- - - - 1. **Synergy Investigation**


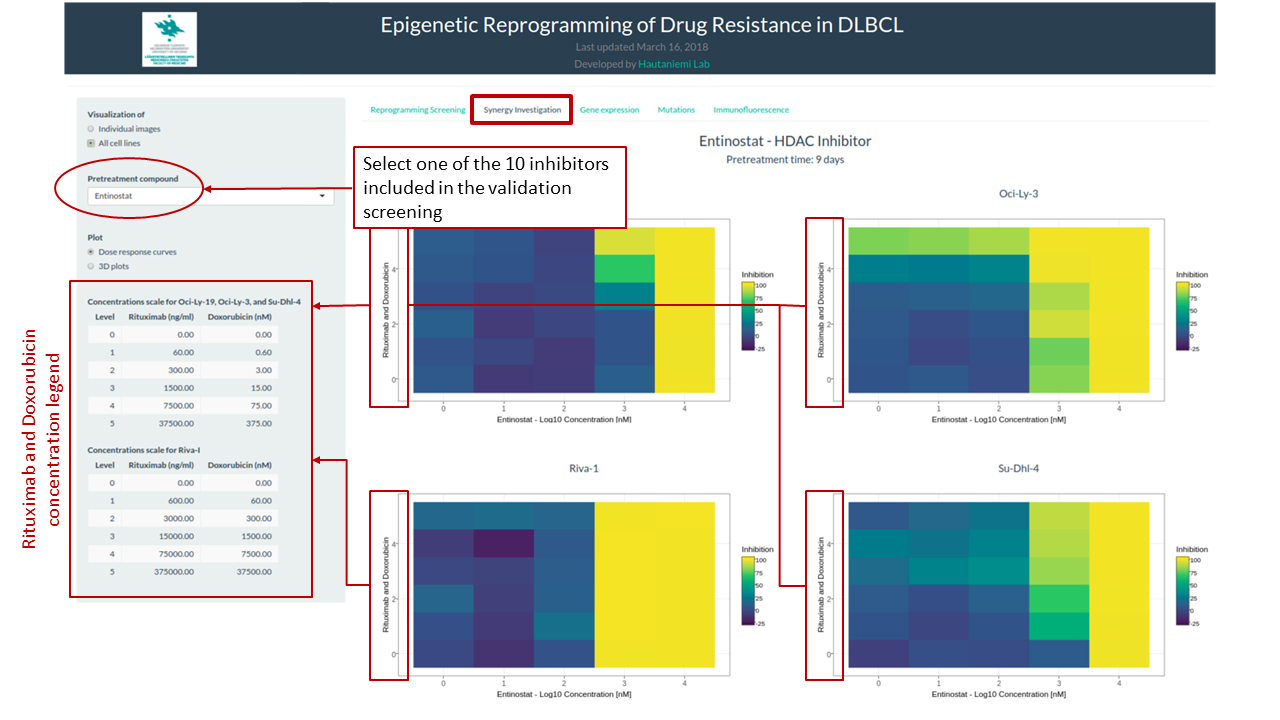
The "Synergy Investigation" tab shows the results of the 9-days synergy assay with five concentrations of the epigenetic inhibitors and five concentrations of rituximab and doxorubicin.

- - - - 1. **Gene Expression**


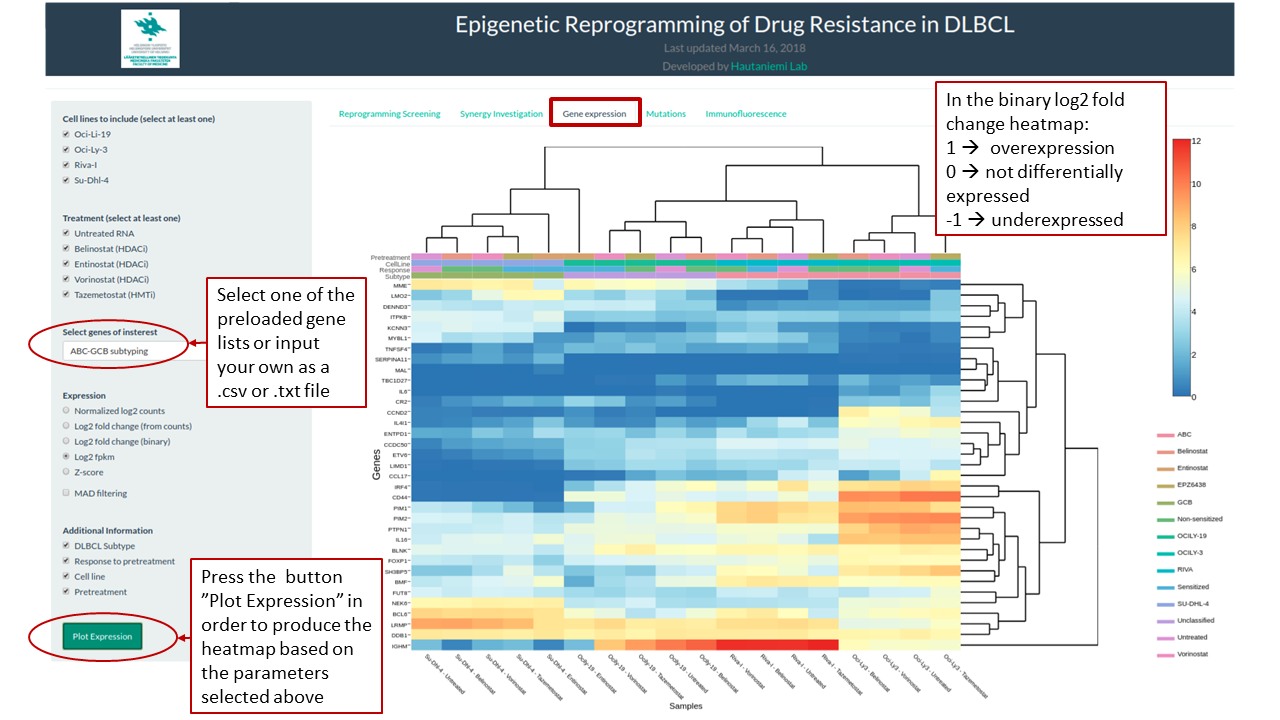
The "Gene expression" tab allows to compare the expression of a pre-selected set of genes across cell lines that are untreated or treated with epigenetic compounds, to access how the reprogramming affected the transcriptome. Some gene sets are preloaded, but the user can load new sets for customized analyses.

- - - - 1. **Mutations**


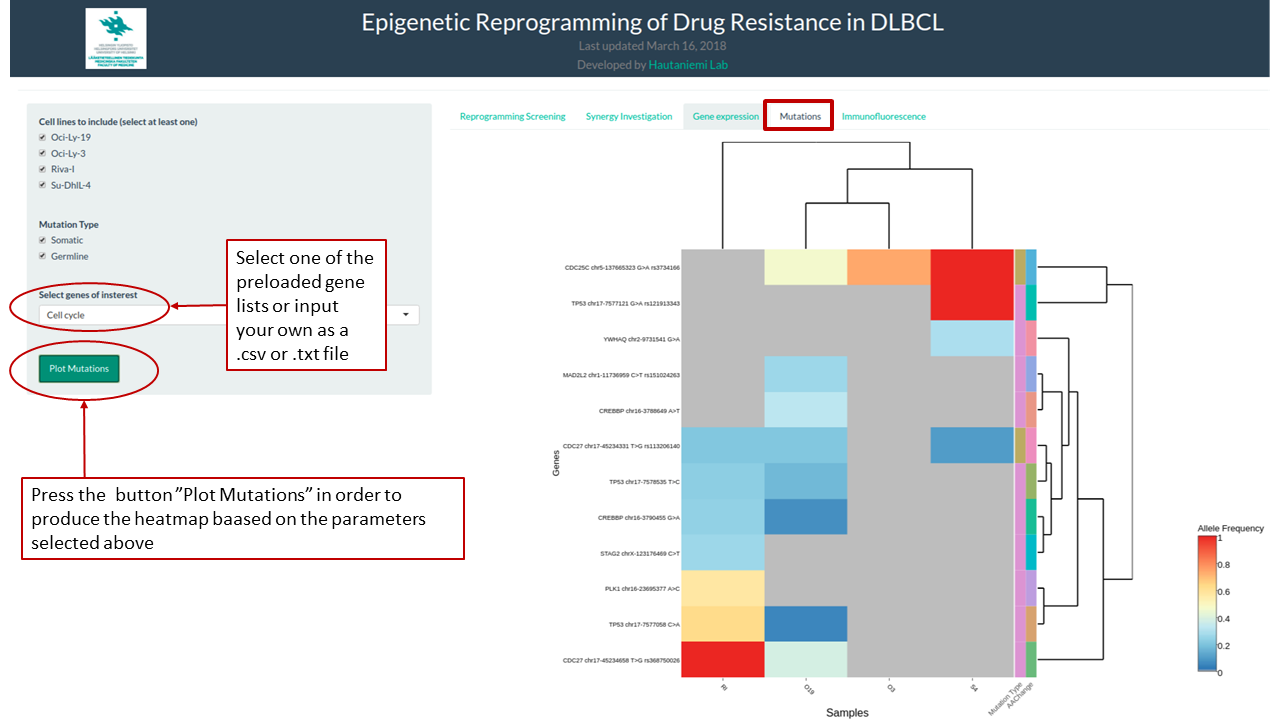
The "Mutations" tab allows to visualize which genes carry mutations in the cell lines included in this study. Some gene sets are preloaded, but the user can load new sets for customized analyses.

- - - - 1. **Immunofluorescence**


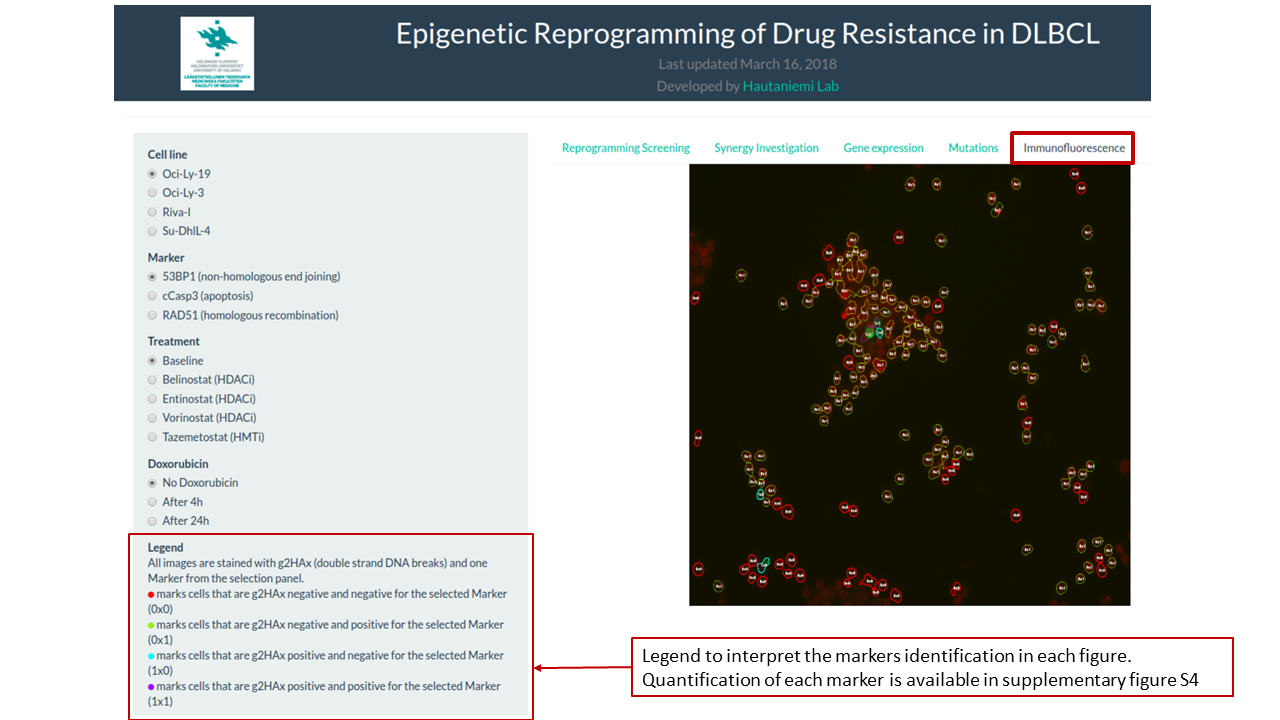
The "Immunofluorescence" tab allows to visualize the images obtained through the immunofluorescence assay used to investigate DNA damage and repair.

**References**

1. Ovaska K, Laakso M, Haapa-Paananen S, Louhimo R, Chen P, Aittomaki V, et al. Large-scale data integration framework provides a comprehensive view on glioblastoma multiforme. Genome Med. 2010;2:65.

2. Zhang XD. Illustration of SSMD, z score, SSMD*, z* score, and t statistic for hit selection in RNAi high-throughput screens. J Biomol Screen. 2011;16:775–85.

3. Ianevski A, He L, Aittokallio T, Tang J. SynergyFinder: a web application for analyzing drug combination dose-response matrix data. Bioinformatics. 2017;33:2413–5.

4. Yadav B, Wennerberg K, Aittokallio T, Tang J. Searching for drug synergy in complex dose-response landscapes using an interaction potency model. Comput Struct Biotechnol J. 2015;13:504–13.

5. Andrews S. FastQC: a quality control tool for high throughput sequence data [Internet]. Available from: http://www.bioinformatics.babraham.ac.uk/projects/fastqc

6. Bolger AM, Lohse M, Usadel B. Trimmomatic: a flexible trimmer for Illumina sequence data. Bioinformatics. 2014;30:2114–20.

7. Li H, Durbin R. Fast and accurate long-read alignment with Burrows-Wheeler transform. Bioinformatics. England; 2010;26:589–95.

8. Van der Auwera GA, Carneiro MO, Hartl C, Poplin R, Del Angel G, Levy-Moonshine A, et al. From FastQ data to high confidence variant calls: the Genome Analysis Toolkit best practices pipeline. Curr Protoc Bioinforma [Internet]. Hoboken, NJ, USA: John Wiley & Sons, Inc.; 2013 [cited 2017 Nov 14];43:11.10.1-33. Available from: http://doi.wiley.com/10.1002/0471250953.bi1110s43

9. Wang K, Li M, Hakonarson H. ANNOVAR: functional annotation of genetic variants from high-throughput sequencing data. Nucleic Acids Res [Internet]. Oxford University Press; 2010 [cited 2017 Nov 14];38:e164. Available from: http://www.ncbi.nlm.nih.gov/pubmed/20601685

10. Forbes SA, Bhamra G, Bamford S, Dawson E, Kok C, Clements J, et al. The Catalogue of Somatic Mutations in Cancer (COSMIC). Curr Protoc Hum Genet [Internet]. Europe PMC Funders; 2008 [cited 2017 Nov 14];Chapter 10:Unit 10.11. Available from: http://www.ncbi.nlm.nih.gov/pubmed/18428421

11. Landrum MJ, Lee JM, Riley GR, Jang W, Rubinstein WS, Church DM, et al. ClinVar: Public archive of relationships among sequence variation and human phenotype. Nucleic Acids Res. 2014;42:D980-985.

12. Griffith M, Spies NC, Krysiak K, McMichael JF, Coffman AC, Danos AM, et al. CIViC is a community knowledgebase for expert crowdsourcing the clinical interpretation of variants in cancer. Nat Genet [Internet]. 2017;49:170. Available from: http://www.nature.com/doifinder/10.1038/ng.3774

13. Cotto KC, Wagner AH, Feng Y-Y, Kiwala S, Coffman AC, Spies G, et al. DGIdb 3.0: a redesign and expansion of the drug–gene interaction database. Nucleic Acids Res. 2017;46:D1068–73.

14. Wishart DS, Feunang YD, Guo AC, Lo EJ, Marcu A, Grant JR, et al. DrugBank 5.0: A major update to the DrugBank database for 2018. Nucleic Acids Res. 2018;46:D1074–82.

15. Whirl-Carrillo M, McDonagh EM, Hebert JM, Gong L, Sangkuhl K, Thorn CF, et al. Pharmacogenomics knowledge for personalized medicine. Clin Pharmacol Ther. 2012;92:414–7.

16. Tamborero D, Rubio-Perez C, Deu-Pons J, Schroeder MP, Vivancos A, Rovira A, et al. Cancer Genome Interpreter annotates the biological and clinical relevance of tumor alterations. Genome Med. 2018;10:25:1–8.

17. Fokkema IFAC, Taschner PEM, Schaafsma GCP, Celli J, Laros JFJ, den Dunnen JT. LOVD v.2.0: The next generation in gene variant databases. Hum Mutat. 2011;32:557–63.

18. Olivier M, Eeles R, Hollstein M, Khan MA, Harris CC, Hainaut P. The IARC TP53 database: New online mutation analysis and recommendations to users. Hum Mutat. 2002;19:607–14.

19. Andrews S. FastQC: a quality control tool for high throughput sequence data.

20. Dobin A, Davis CA, Schlesinger F, Drenkow J, Zaleski C, Jha S, et al. STAR: ultrafast universal RNA-seq aligner. Bioinformatics. 2013;29:15–21.

21. Roberts A, Pachter L. Streaming fragment assignment for real-time analysis of sequencing experiments. Nat Methods. 2013;10:71–3.

22. Love MI, Huber W, Anders S. Moderated estimation of fold change and dispersion for RNA-seq data with DESeq2. Genome Biol. 2014;15:550.

23. Kuleshov M V, Jones MR, Rouillard AD, Fernandez NF, Duan Q, Wang Z, et al. Enrichr: a comprehensive gene set enrichment analysis web server 2016 update. Nucleic Acids Res. 2016;44:W90--97.

24. Kanehisa M, Furumichi M, Tanabe M, Sato Y, Morishima K. KEGG: New perspectives on genomes, pathways, diseases and drugs. Nucleic Acids Res. 2017;45:D353–61.

25. Slenter DN, Kutmon M, Hanspers K, Riutta A, Windsor J, Nunes N, et al. WikiPathways: A multifaceted pathway database bridging metabolomics to other omics research. Nucleic Acids Res. 2018;46:D661-667.

26. Croft D, O’Kelly G, Wu G, Haw R, Gillespie M, Matthews L, et al. Reactome: A database of reactions, pathways and biological processes. Nucleic Acids Res. 2011;39:D691–7.

27. Hänzelmann S, Castelo R, Guinney J. GSVA: Gene set variation analysis for microarray and RNA-Seq data. BMC Bioinformatics. 2013;

28. Tumiati M, Munne PM, Edgren H, Eldfors S, Hemmes A, Kuznetsov SG. Rad51c- and Trp53-double-mutant mouse model reveals common features of homologous recombination-deficient breast cancers. Oncogene. 2016;35:4601–10.

29. Rantanen V, Valori M, Hautaniemi S. Anima: modular workflow system for comprehensive image data analysis. Front Bioeng Biotechnol. 2014;2:25.

30. Chang W, Cheng J, Allaire JJ, Xie Y, McPherson J. shiny: Web Application Framework for R. 2018.

31. Sievert C. plotly for R. 2018.

32. Galili T, O’Callaghan A, Sidi J, Sievert C. Heatmaply: An R package for creating interactive cluster heatmaps for online publishing. Bioinformatics. 2018;34:1600–2.

33. Solomon DA, Kim T, Diaz-Martinez LA, Fair J, Elkahloun AG, Harris BT, et al. Mutational inactivation of STAG2 causes aneuploidy in human cancer. Science (80- ). 2011;333:1039–43.

34. Evers L, Perez-Mancera PA, Lenkiewicz E, Tang N, Aust D, Knösel T, et al. STAG2 is a clinically relevant tumor suppressor in pancreatic ductal adenocarcinoma. Cancer Cell Signal Target Signal Pathways Towar Ther Approaches to Cancer. 2014;6:9.

35. Wu R, Nie Q, Tapper EE, Jerde CR, Dunlap GS, Shrestha S, et al. Histone H3K27 trimethylation modulates 5-fluorouracil resistance by inhibiting PU.1 binding to the DPYD promoter. Cancer Res. 2016;

36. Kolukula VK, Sahu G, Wellstein A, Rodriguez OC, Preet A, Iacobazzi V, et al. SLC25A1, or CIC, is a novel transcriptional target of mutant p53 and a negative tumor prognostic marker. Oncotarget. 2014;5:1212–25.

37. Krupa R, Sliwinski T, Wisniewska-Jarosinska M, Chojnacki J, Wasylecka M, Dziki L, et al. Polymorphisms in RAD51, XRCC2 and XRCC3 genes of the homologous recombination repair in colorectal cancer-a case control study. Mol Biol Rep. 2011;38:2849–54.

38. Manandhar M, Boulware KS, Wood RD. The ERCC1 and ERCC4 (XPF) genes and gene products. Gene. 2015;569:153–61.

39. Jiang Y, Soong TD, Wang L, Melnick AM, Elemento O. Genome-wide detection of genes targeted by Non-Ig somatic hypermutation in lymphoma. PLoS One. 2012;7:e40332.

40. Allione A, Guarrera S, Russo A, Ricceri F, Purohit R, Pagnani A, et al. Inter-individual variation in nucleotide excision repair pathway is modulated by non-synonymous polymorphisms in ERCC4 and MBD4 genes. Mutat Res - Fundam Mol Mech Mutagen. 2013;751–752:49–54.
